# Translating algorithmic fairness into health practice: formative evaluation of the fairness-to-action framework

**DOI:** 10.3389/frai.2026.1911214

**Published:** 2026-09-21

**Authors:** Sara Altamirano, Daniel Anadria, Stefanie van der Wees, Paulien Tensen, Steven van de Vijver, Sennay Ghebreab

**Affiliations:** 1 Informatics Institute, Faculty of Science, University of Amsterdam, Amsterdam, Netherlands; 2 Department of Methodology and Statistics, Faculty of Social and Behavioral Sciences, Utrecht University, Utrecht, Netherlands; 3 Julius Center for Health Sciences and Primary Care, University Medical Center Utrecht, Utrecht University, Utrecht, Netherlands; 4 Department of Engineering Systems and Services, Faculty of Technology, Policy and Management, Delft University of Technology, Delft, Netherlands; 5 Department of Public and Occupational Health, Amsterdam Public Health, Amsterdam UMC Location University of Amsterdam, Amsterdam, Netherlands; 6 Amsterdam Health & Technology Institute, Amsterdam, Netherlands; 7 Department of General Practice, OLVG Hospital, Amsterdam, Netherlands

**Keywords:** AI governance, algorithmic fairness, digital health, formative evaluation, health equity, knowledge translation, responsible AI, stakeholder mapping

## Abstract

**Introduction:**

Algorithmic fairness is increasingly acknowledged as a critical concern in digital health; however, existing knowledge on fairness remains challenging to operationalize and systematically embed into routine practice. Our study examines the knowledge-practice gap through a formative evaluation of the Fairness-to-Action framework and the development of a guidance artifact.

**Methods:**

The study was conducted within HealthEmove, a digital health initiative focused on electronic personal health records for mobile populations. Using an exploratory mixed-methods design, we interviewed 11 professional stakeholders using all 22 Fairness-to-Action prompts. We also collected prompt-level Clarity, Relevance, and Usability ratings and conducted stakeholder responsibility mapping.

**Results:**

Qualitative findings suggest that fairness knowledge was available in HealthEmove but unevenly distributed across roles, partially formalized, and not yet embedded in shared routines. Stakeholders' self-reported ratings were concentrated at the upper end of the Clarity, Relevance, and Usability scales and were interpreted as formative indicators of perceived fit within this case. Responsibility mapping made cross-role dependencies explicit and informed a proof-of-concept guidance artifact for the Junior Researcher role. The artifact underwent formative assessment by two of the 11 participants: the target role holder and one closely collaborating respondent. Their preliminary feedback suggests that clearer documentation expectations and onboarding may improve use.

**Discussion:**

This single-case study contributes a formative evaluation of the Fairness-to-Action framework and illustrates a procedure for turning its prompts into role-specific guidance within an early-stage digital health project. At the process level, it examines the fairness knowledge-practice gap by treating fairness knowledge as a resource that must be accessed, interpreted, and operationalized across project roles, rather than only specified as a technical or governance requirement.

## 1 Introduction

Algorithmic systems increasingly inform health risk identification, resource allocation, and healthcare delivery (Paulus and Kent, 2020; Morgenstern et al., 2020; Fletcher et al., 2021). Algorithmic fairness, understood here as concern with whether such systems produce or sustain systematic disadvantage across groups and contexts (Mehrabi et al., 2021), has therefore become a major concern in artificial intelligence (AI) research and digital health practice. Proposed technical, documentation, and oversight-oriented approaches include fairness metrics, reporting guidelines, and auditing methods (Dwork et al., 2012; Hardt et al., 2016; Kusner et al., 2017; Barocas et al., 2023; Mitchell et al., 2019; Raji et al., 2020; World Health Organization, 2021; Collins et al., 2024). Yet prior work shows that adopting these approaches often remains difficult because fairness work depends on task definition, organizational support, role responsibility, and governance over time (Veale et al., 2018; Selbst et al., 2019; Holstein et al., 2019; Jobin et al., 2019; Madaio et al., 2020; Rakova et al., 2021). In practice, teams must determine what fairness means in context, which fairness knowledge is relevant, who has authority to act, and how fairness concerns should be monitored. Algorithmic fairness is therefore both a complex technical problem and a knowledge translation and coordination challenge.

This gap between available fairness knowledge and coordinated project work can be consequential in digital health, where fairness risks can emerge across the health AI lifecycle, particularly when data reflect structural inequities or underrepresented marginalized groups, or when models are developed from historically biased or incomplete data (Gianfrancesco et al., 2018; Rajkomar et al., 2018; Obermeyer et al., 2019; Wiens et al., 2019; Suresh and Guttag, 2021). Digital health infrastructures can also reproduce or exacerbate disparities when differences in access, digital literacy, and institutional conditions are not addressed (Latulippe et al., 2017; Van Dijk, 2020; Bober et al., 2024). These risks are especially salient in high-resource settings such as the Netherlands, where advanced digital health infrastructures and AI capacity coexist with persistent inequalities among ethnic minorities and socioeconomically disadvantaged groups (Ikram et al., 2014; Kroneman et al., 2016; Ilozumba et al., 2022). Accordingly, digital health provides a suitable setting for examining how fairness knowledge is translated into project work.

To examine this problem empirically, our study builds on previous work that framed the limited uptake of algorithmic fairness in public health as a knowledge–practice gap (Altamirano et al., 2026). Drawing on complexity-informed health services research, we treat digital health projects as settings where knowledge must be adapted to local roles, workflows, and constraints (Greenhalgh and Papoutsi, 2018). Rather than treating fairness only as a technical property of models, we focus on how fairness-related knowledge becomes actionable in project work. We examine Fairness-to-Action (F2A) as a practice-facing set of structured prompts for organizing fairness knowledge and project-level responsibilities in an applied digital health setting. We ask: *How can the Fairness-to-Action framework be formatively evaluated and instantiated within a digital health project to develop role-specific guidance for algorithmic fairness?*

Our study is conducted within the *HealthEmove* initiative^1^ (Van de Vijver et al., 2023), which collaborates with the Dutch program *De Digitale Voordeur* (Digital Front Door; DFD), a municipal care and welfare platform in Amsterdam.^2^ HealthEmove is being redesigned as an app-based electronic personal health record (EPHR) for release through app stores. It is intended as a portable, patient-centered record^3^ that can travel with individuals across care contexts. The app aims to support equitable access to health records, continuity of care, and patient agency for mobile populations, defined here as people who move across local, national, or international contexts while facing disadvantaged circumstances, including refugees, asylum seekers, and undocumented migrants (McAuliffe and Oucho, 2024). Because the initiative is in an early deployment and redesign phase involving multiple stakeholders and evolving governance arrangements, it provides a suitable context to examine fairness as a project-level coordination and knowledge translation problem before large-scale use.

Our study makes three contributions. First, it empirically applies the F2A framework across roles within an applied digital health project, generating empirical evidence on how its prompts are interpreted in relation to project practice. Second, it provides a formative evaluation of the framework through prompt-level numerical assessment of Clarity, Relevance, and Usability. Third, it develops and applies a responsibility-mapping instantiation procedure through a proof-of-concept application to one role. Overall, within this single formative case, this study contributes to the process-level operationalization of algorithmic fairness by illustrating a route from F2A prompts to role-specific guidance as a possible response to the gap between fairness knowledge and project-level practice. Because role differentiation, distributed responsibilities, and coordination needs may also arise in other multi-stakeholder settings, this single formative case provides an initial process-level basis for examining the approach's potential analytical transferability beyond digital health, subject to context-specific evaluation and instantiation.

## 2 Related work

Efforts to operationalize algorithmic fairness and AI ethics in practice have produced documentation artifacts, auditing processes, governance frameworks, and domain-specific standards. Documentation tools provide structured reporting of model performance, intended use, and limitations (Mitchell et al., 2019) and extend to dataset-level transparency (Gebru et al., 2021), while checklist-based approaches structure fairness considerations during system design and development (Madaio et al., 2020). In healthcare, domain-specific frameworks further identify sources of algorithmic bias and mitigation strategies across AI development and deployment (Agarwal et al., 2023). Auditing and implementation review frameworks introduce systematic evaluation of system behavior and risks prior to deployment, while also raising questions about audit standards, disclosure, stakeholder involvement, and follow-up mechanisms (Raji et al., 2020; Costanza-Chock et al., 2022; Wells et al., 2025). Governance-oriented work maps and synthesizes ethical principles across AI guidance documents (Jobin et al., 2019; Mittelstadt, 2019; Hagendorff, 2020) and, in some cases, translates them into more actionable guidance, standards, and organizational governance processes for responsible AI development and use (Morley et al., 2020; World Health Organization, 2021; Mäntymäki et al., 2022; IEEE, 2025; Lu et al., 2024). In healthcare, reporting standards provide structured guidance for transparent and complete reporting of AI systems (Liu et al., 2020; Hernandez-Boussard et al., 2020; Sounderajah et al., 2021; Collins et al., 2024), while dedicated assessment tools evaluate model quality, risk of bias, and applicability (Moons et al., 2025). Recent frameworks further address clinical AI governance, trustworthy deployment, pre-implementation review, post-implementation monitoring, and workflow-embedded ethics guidance (Liao et al., 2022; Lekadir et al., 2025; Wells et al., 2025; Nemteanu et al., 2026). These approaches specify what should be documented, evaluated, and governed, but often assume that fairness knowledge can be accessed, interpreted, and operationalized within project workflows.

Accordingly, subsequent work has examined how such frameworks are implemented and used in practice, focusing on feasibility, usability, and integration into real-world settings. In human–AI interaction and human-centered machine learning, studies assess how users engage with explanations, interfaces, and collaborative systems, focusing on interpretability, trust, perceived capability, human-centered evaluation, and healthcare decision support (Amershi et al., 2019; Cai et al., 2019a,b; Sperrle et al., 2021; Lai et al., 2021). In healthcare, evaluation frameworks examine adoption, utility, translational value, and implementation within clinical workflows (Reddy et al., 2021; Gama et al., 2022; Glasgow et al., 2019), often drawing on implementation science approaches that emphasize contextual determinants, feasibility, uptake, and sustainability in practice (Damschroder et al., 2009; Nilsen, 2015; Proctor et al., 2011). Complementary research highlights how problem formulation, organizational constraints, and complexity in real-world health service settings shape how such tools are interpreted and used (Passi and Barocas, 2019; Greenhalgh and Papoutsi, 2018; Veale et al., 2018), while methods such as ECCOLA are evaluated for their ability to support ethical reflection in development processes (Vakkuri et al., 2021). Across much of this work, evaluation primarily focuses on whether tools can be adopted and integrated into workflows. However, these approaches place limited focus on whether fairness-related knowledge is interpretable and actionable across project roles before it is embedded in tools, workflows, or governance processes. This limitation aligns with recent work indicating that AI ethics evaluation remains fragmented across principles, system components, and harm types, with limited systems-level integration (Rismani et al., 2025).

A knowledge translation perspective situates these limitations within research on moving research-based knowledge into practice. Foundational work conceptualizes knowledge translation and implementation as context-dependent processes shaped by stakeholder interaction, organizational conditions, actor roles, and system dynamics (Graham et al., 2006; Damschroder et al., 2009; Grimshaw et al., 2012). Subsequent work evaluates implementation through acceptability, adoption, feasibility, and sustainability (Proctor et al., 2011) and examines usability and contextual fit as upstream determinants of uptake (Lyon et al., 2021). Knowing-doing gap research suggests that organizations may fail to translate available knowledge into action despite its availability (Pfeffer and Sutton, 2000). This literature primarily examines whether tools can be adopted and used but less directly addresses whether the knowledge required to use them is accessible, interpretable across roles, and translatable into project-level responsibilities before implementation. Consequently, less is known about how fairness frameworks function as role-sensitive knowledge translation instruments, particularly how their guidance is interpreted and converted into situated project work.

## 3 Conceptual background

### 3.1 Fairness as a knowledge translation problem

Despite advances in fairness definitions, measurement approaches, and mitigation strategies, gaps persist between awareness of fairness concerns and their integration into project practice. Empirical studies indicate that practitioners recognize fairness issues but struggle to operationalize them within existing workflows, institutional constraints, and decision structures (Veale et al., 2018; Rakova et al., 2021). Principles, audits, and review processes are also difficult to embed in organizational routines without structures for accountability, coordination, and follow-up (Ananny and Crawford, 2018; Ali et al., 2023; Raji et al., 2020), particularly in healthcare settings that require alignment with safety, equity, transparency, and ongoing monitoring (World Health Organization, 2021; Wells et al., 2025).

These challenges reflect a broader knowledge–practice gap, where knowledge exists but is not routinely connected to action (Graham et al., 2006; Grimshaw et al., 2012; Pfeffer and Sutton, 2000). In algorithmic systems, this gap is intensified by the need to translate high-level goals into operational decisions such as defining target variables, selecting proxies, and structuring problem formulations, which are discretionary, context-dependent, and shaped by assumptions about how constructs are operationalized (Jacobs and Wallach, 2021; Passi and Barocas, 2019). As a result, fairness-related considerations often remain implicit, fragmented, or weakly connected to everyday decision-making.

Our study conceptualizes algorithmic fairness as a knowledge translation problem, focusing on how fairness-related knowledge becomes usable in project work. Knowledge translation research indicates that movement from knowledge to action depends on context, roles, and implementation processes rather than availability alone (Graham et al., 2006; Damschroder et al., 2009; Nilsen, 2015). Within this perspective, translation refers to how fairness-related knowledge, including definitions, metrics, guidelines, and procedural expectations, is accessed, interpreted, and operationalized within role-specific tasks and decisions.

### 3.2 The fairness-to-action framework

The F2A framework (Altamirano et al., 2026) was developed to examine where the translation of algorithmic fairness knowledge stalls before reaching practice. It was introduced in prior work on ML-driven public health research, where empirical findings on fairness perceptions and practices were mapped onto three research–practice gap lenses: the Knowledge–Practice Gap, the Knowledge–to–Action Cycle, and the Knowing–Doing Gap. In that work, F2A was positioned as an evidence-informed diagnostic scaffold rather than a prescriptive or predictive model. It does not define fairness criteria, select fairness metrics, or evaluate model performance. Instead, it organizes questions about how fairness-related knowledge is defined, accessed, transferred, implemented, monitored, and sustained.

F2A is structured around three linked dimensions: *What, How*, and *Why* (Figure 1). The *What* dimension corresponds to the Knowledge–Practice Gap and examines the current state of fairness-related knowledge: what knowledge exists, who holds it, where it comes from, how it is accessed, and with what effect. The *How* dimension corresponds to the Knowledge–to–Action Cycle and examines how fairness-related knowledge moves through knowledge creation and action processes, including inquiry, synthesis, tools, needs identification, knowledge selection, adaptation to context, barrier assessment, implementation, monitoring, evaluation, and sustainment. The *Why* dimension corresponds to the Knowing–Doing Gap and examines mechanisms that may explain why translation stalls, including whether fairness remains narrowly framed, whether discussion substitutes for action, whether routine reporting replaces learning, whether operational concerns limit disclosure or corrective action, whether accuracy metrics outweigh broader fairness judgments, and whether disciplinary divides slow collective work.

**Figure 1:**
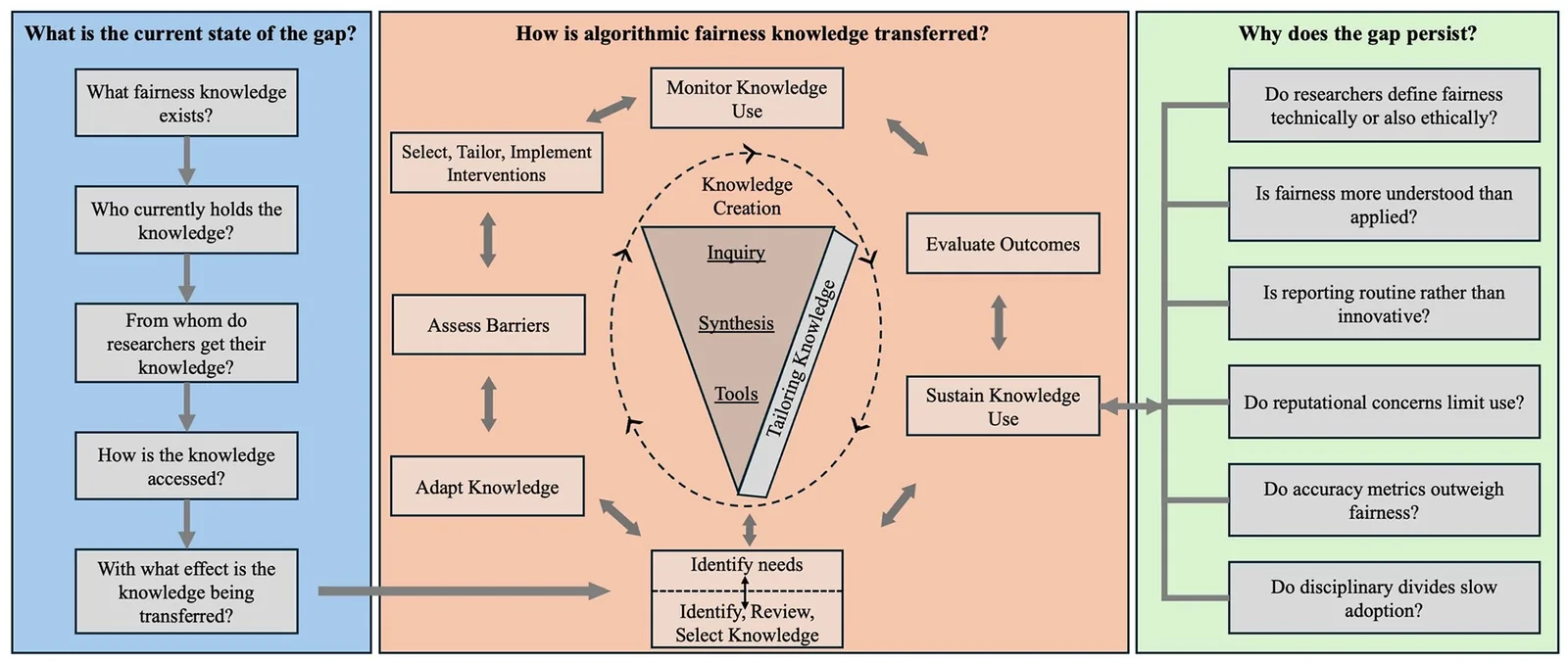
Fairness-to-Action (F2A) framework (reproduced from Altamirano et al., 2026; Source: https://ojs.aaai.org/index.php/IASEAI/article/view/43011). The framework structures algorithmic fairness as a knowledge translation process across three dimensions. The *What* dimension examines the current state of fairness-related knowledge, including what knowledge exists, who holds it, how it is accessed, and with what effect. The *How* dimension examines how fairness-related knowledge moves through knowledge creation and action processes. The *Why* dimension examines why translation stalls, including organizational and systemic mechanisms that can prevent fairness-related knowledge from becoming routine project practice. In the present study, this original framework structure is used to develop stakeholder prompts, support formative evaluation through Clarity, Relevance, and Usability assessment, and guide responsibility mapping into role-specific guidance.

This structure treats algorithmic fairness as a methodological, organizational, and systemic problem. Methodologically, fairness knowledge may be inconsistently defined or weakly formalized. Organizationally, knowledge may flow through informal or external channels rather than stable internal structures. Systemically, incentives, reporting routines, performance pressures, and disciplinary boundaries may prevent fairness knowledge from becoming sustained practice. F2A therefore supports analysis of the conditions under which fairness-related knowledge becomes actionable, rather than assuming that the existence of fairness guidance is sufficient for practice change.

In the present study, F2A is used both as an analytical framework for organizing stakeholder responses and as the object of formative evaluation through assessment of the Clarity, Relevance, and Usability of its prompts across roles. We use the framework to examine whether its diagnostic questions can be interpreted and operationalized within a live digital health project and to support responsibility mapping into role-specific guidance. See Section 5 for the condensed formulation of the F2A prompts and Supplementary material C for the full questionnaire.

### 3.3 Stakeholder responsibility mapping

Fairness-related tasks in AI projects are distributed across roles with different levels of authority, involvement, and expertise. Prior research shows that fairness and accountability challenges are shaped by organizational conditions and the absence of clear coordination mechanisms (Veale et al., 2018; Rakova et al., 2021), while key decisions affecting fairness are negotiated across roles without explicit responsibility structures (Passi and Barocas, 2019). Similar concerns appear in healthcare and governance guidance calling for clearer allocation of review, monitoring, and oversight responsibilities (World Health Organization, 2021; Wells et al., 2025; IEEE, 2025).

To address this, the study uses the Responsible, Accountable, Consulted, and Informed (RACI) framework, a widely used project management tool for clarifying role responsibilities, task ownership, accountability, consultation, and information flows (Brower et al., 2021; Project Management Institute, 2021). RACI identifies who executes tasks, who holds accountability, and which roles should be consulted or informed. This role-based mapping also aligns with governance work that emphasizes explicit decision rights, responsibility allocation, and the institutionalization of ethics-related responsibilities within organizations (Kofman et al., 2009; Metcalf et al., 2019). Here, RACI is applied with stakeholder input to map fairness-related tasks derived from the F2A framework to specific roles, enabling assessment of whether these expectations are actionable within existing role structures and supporting the translation of F2A prompts into role-specific checklists.

## 4 Materials and methods

### 4.1 Study overview

This exploratory mixed-methods single-case study conducted a formative evaluation and role-based instantiation of F2A within an applied digital health project. The formative evaluation assessed stakeholder interpretations of F2A prompts and prompt-level Clarity, Relevance, and Usability (CRU) ratings. The role-based instantiation examined how selected F2A tasks could connect to project responsibilities through RACI-based responsibility mapping. The study therefore assessed whether F2A can function as a practice-facing knowledge translation instrument, rather than evaluating downstream fairness outcomes, intervention effectiveness, or psychometric measurement properties.

The design integrated three components: (1) role-stratified stakeholder interviews structured around all 22 F2A prompts, generating open-ended responses on fairness-related practices; (2) item-level CRU ratings for each prompt; and (3) RACI-based responsibility mapping to situate fairness-related tasks within project roles and decision authority. These components were analyzed separately. Qualitative interview responses were used to examine how fairness-related knowledge is accessed, interpreted, and operationalized across roles; CRU ratings were analyzed descriptively to assess prompt-level clarity, relevance, and usability; and RACI mapping supported role-based framework instantiation.

Formative evaluation here denotes assessment of the F2A prompts' practical interpretability and perceived fit with roles and workflows. It includes qualitative assessment of participants' interpretations and responses, and descriptive quantitative assessment of their perceived understandability, relevance to participants' responsibilities, and usability within existing project workflows. CRU scores provide self-reported item-level indicators of these dimensions.

This study draws on a socio-technical, implementation-oriented perspective emphasizing sensemaking, feasibility, and coordination rather than outcome measurement (Trist and Bamforth, 1951; Bostrom et al., 2009; Greenhalgh and Papoutsi, 2018). Consistent with early-stage implementation-focused evaluation (Bowen et al., 2009; Proctor et al., 2011), our approach prioritizes contextual practical usefulness rather than formal measurement properties. Figure 2 summarizes the study design, including data inputs and preparation, analytic procedures, and outputs.

**Figure 2:**
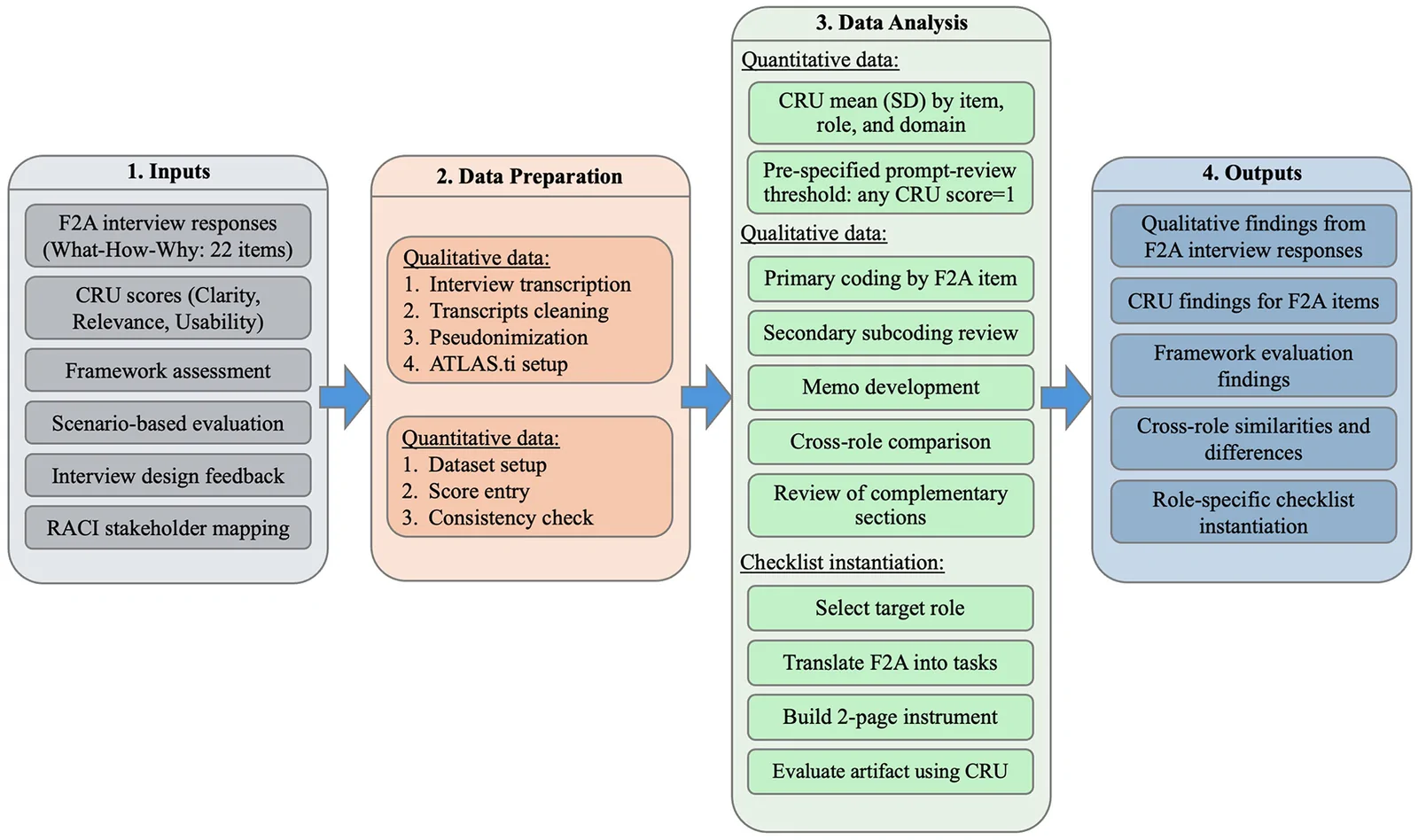
Overview of the study workflow from data inputs to analytic outputs. Multiple data sources were prepared for quantitative and qualitative analyses, alongside a role-specific checklist instantiation procedure. Analyses generated the principal findings reported in the Results section, including findings from the formative evaluation of F2A, cross-role patterns, and a role-specific implementation artifact. F2A, Fairness-to-Action; CRU, Clarity, Relevance, Usability; RACI, Responsible, Accountable, Consulted, Informed; SD, standard deviation.

### 4.2 Empirical setting and context

Our study is conducted within the *HealthEmove* initiative, an applied digital health project led by the Amsterdam Health & Technology Institute with academic, community, healthcare, and technology partners. HealthEmove is developing an app-based EPHR to support continuity of care, patient agency, and equitable access for mobile populations, including refugees, asylum seekers, and undocumented migrants (Van de Vijver et al., 2023). The initiative previously used software from the UK-based company Patients Know Best (Tensen et al., 2025a), but since 2025 has been redesigning its own app with DFD to better fit the target population. This connects HealthEmove to a broader municipal care and welfare platform that aims to place residents more centrally within Amsterdam's care and welfare domain and guide them toward appropriate support across care, welfare, self-help, and social support.^4^ DFD partners include the Municipality of Amsterdam, Zilveren Kruis (health insurer), Sigra (regional healthcare network), A'dam Vitaal en Gezond (Amsterdam public health initiative), Amsterdamse Huisartsen Alliantie (primary care alliance), Amsterdam UMC (university medical center), and MedMij (Dutch framework for personal health data exchange). HealthEmove partners include local NGOs and community-facing care organizations that provide access to the target population, including Kruispost, a voluntary general practice for uninsured residents. Figure 3 summarizes the DFD platform components and affiliated implementation initiatives.

**Figure 3:**
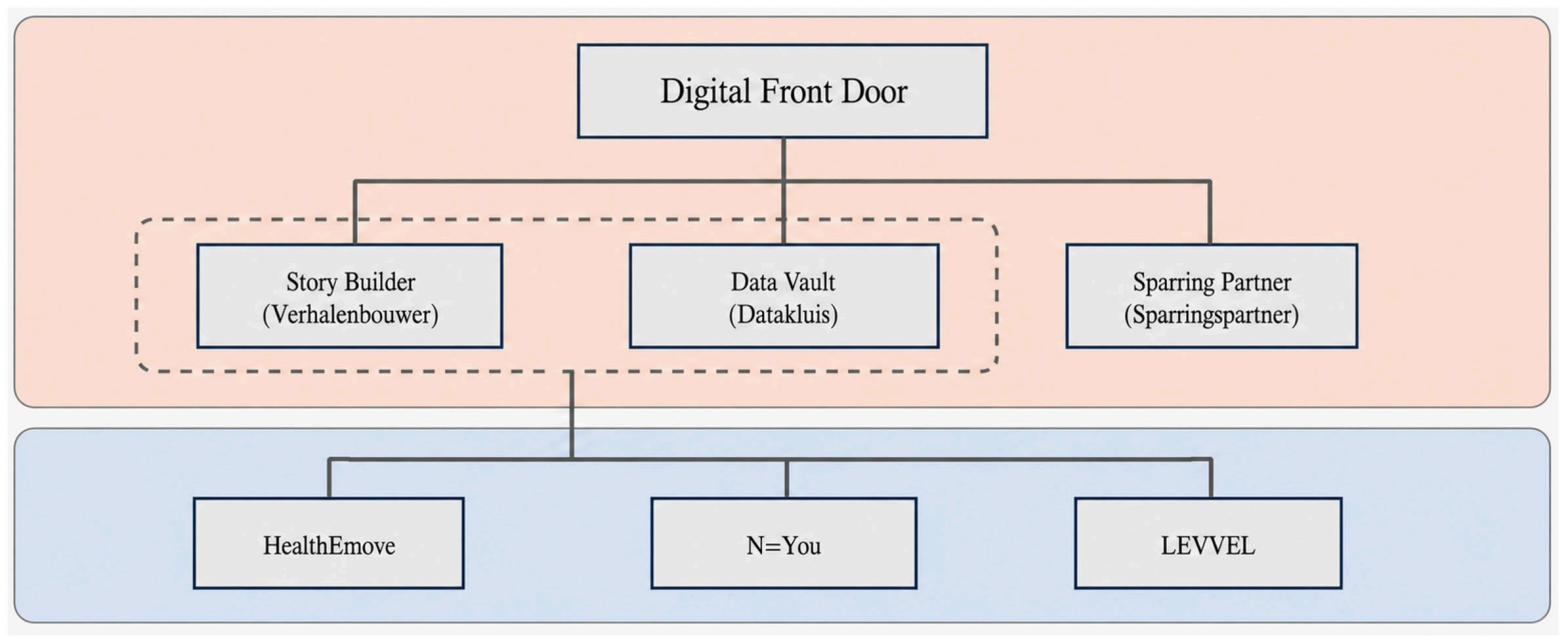
Digital Front Door Amsterdam (DFD) program and implementation ecosystem. DFD is the central municipal care and welfare platform program. Platform and support components include Story Builder (Verhalenbouwer), Data Vault (Datakluis), and Sparring Partner (Sparringspartner). HealthEmove, *N* = You, and LEVVEL are affiliated implementation initiatives. The dashed outline indicates that Story Builder and Data Vault are linked components jointly associated with the affiliated implementation initiatives, whereas Sparring Partner is a separate DFD component not directly related to those initiatives.

Mobile populations often experience fragmented healthcare trajectories, interacting with multiple providers across locations without consistent medical records. This can delay care, duplicate procedures, and reduce the quality of clinical decision-making. EPHRs may address these challenges by enabling individuals to access, manage, and share their health information across contexts, thereby supporting informational continuity and coordinated care. However, evidence identifies persistent implementation barriers, including limited digital and health literacy in target populations, concerns about data security and privacy, and challenges in user adoption and sustained use (Tensen et al., 2025a,b).

Within the Netherlands, digital health infrastructures are relatively advanced; however, fragmentation across electronic health record systems and strict data protection regulations constrain data sharing across providers (Tensen et al., 2025b). These constraints also make data stewardship important, since responsible reuse depends on data and related research objects being findable, accessible under appropriate conditions, interoperable, and reusable (Wilkinson et al., 2016). For mobile populations, and particularly undocumented migrants, these constraints are compounded by limited system access and structural barriers to care, resulting in incomplete or unavailable medical histories and reduced continuity of care (Tensen et al., 2025b,c). A small qualitative study with Dutch healthcare providers (*N* = 13) found generally positive attitudes toward EPHRs for undocumented migrants, while also identifying key preconditions for use, including the establishment of trust between patients and providers, strong data security guarantees, patient control over data, and, for some participants, integration into existing workflows (Tensen et al., 2025c).

HealthEmove builds on these insights and is currently implemented as a pilot in the Netherlands, while being conceptually oriented toward broader applicability across settings involving mobile populations living in disadvantaged circumstances (Tensen et al., 2025b; Van de Vijver et al., 2023). Existing research on EPHRs emphasizes that mobility-related care fragmentation occurs across national and regional boundaries and that solutions must support cross-context data access rather than remain tied to single health systems (Tensen et al., 2025a,b). Accordingly, HealthEmove aligns with broader efforts to develop digital health solutions that support continuity of care for mobile populations across different countries and health systems (Van de Vijver et al., 2023; Tensen et al., 2025a).

At the same time, HealthEmove is currently in an early deployment and redesign phase, involving newly commissioned proprietary interfaces and collaboration with external and internal developers. This stage is characterized by ongoing negotiation of governance arrangements, role boundaries, and decision authority related to data use and AI-enabled functionalities. Studying the project at this stage provides a setting in which fairness-related practices are not yet stabilized, allowing examination of how fairness-related knowledge is accessed, interpreted, and applied before routines and technical architectures become fixed.

### 4.3 Stakeholder sampling and recruitment

Participants were recruited using purposive, role-stratified sampling based on involvement in the design, development, governance, or implementation of the HealthEmove project. This strategy was selected to capture variation in how fairness-related knowledge is accessed, interpreted, and operationalized across distinct functional roles, rather than to achieve statistical representativeness. Inclusion criteria required participants to occupy roles with decision-making, implementation, advisory, or coordination responsibilities relevant to fairness-related tasks, with no exclusion criteria applied beyond role relevance and availability. A total of 11 participants were recruited, covering all major role categories identified in the system mapping, including project leadership, project management, data analysis, software development, user experience and communication, legal and ethics advisory functions, and public health or community partners. No recruited participant withdrew after consent. For this formative case study, sampling was considered sufficient once coverage was achieved across the main HealthEmove roles and directly relevant DFD-facing functions. The recruitment pool comprised 15 role-relevant stakeholders; 11 participated, while the four non-participating stakeholders held adjacent rather than direct HealthEmove roles and represented partially overlapping areas of expertise.

For reporting purposes, *technical roles* refer to participants primarily involved in data analysis, software development, AI engineering, technical product ownership, or IT leadership, whereas *managerial or governance-oriented roles* refer to participants primarily involved in project leadership, strategy, legal or ethics advisory work, public health, or related coordination functions. These groupings are used only for reporting and do not imply homogeneous expertise or responsibilities within each category.

### 4.4 Fairness-to-action interviews and questionnaire data collection

Data were collected using a structured interview questionnaire administered between December 2025 and January 2026. Participants were identified through the HealthEmove network and invited by email or in person. Participants received an information sheet describing the study purpose, researcher contact details, participation format, confidentiality, and data handling before participation. They completed the instrument in their preferred mode, either in written form or through in-person or online video-based interviews. Interviews were conducted by the primary researcher, whose work focuses on algorithmic fairness in public health, using a standardized protocol, audio-recorded with participant consent, and transcribed for analysis. No repeat interviews were conducted, and no non-participants were present during interviews. All responses were collected in English. To maintain consistency across formats, participants received the same questions in the same order regardless of whether they responded in writing, in person, or online. Field notes were recorded where relevant to document contextual observations and follow-up clarifications. Transcripts were not returned to participants for correction. Mode variation was treated as a pragmatic accommodation and not as a source of systematic variation in responses.

The questionnaire comprised three main sections and a short instrument-feedback appendix. Section 1 contained the 22 open-ended F2A items, organized along the *What* (items 1–5), *How* (items 6–16), and *Why* (items 17–22) dimensions, each followed by CRU scores. Section 2 elicited overall assessments of framework structure, perceived completeness, and practical usefulness using 1–5 scores and optional comments. Section 3 presented the Hypothetical Fairness Risk Scenario, derived from a planned HealthEmove feature, and included structured scores and reflections on perceived risks, coordination needs, and responsibility attribution. A final appendix collected feedback on interview questionnaire clarity, usability, workload, and completion time.

Participants were instructed to answer in their own words and, where relevant, to respond hypothetically when a practice or process was not yet in place. They could also indicate when an item did not apply to their role (e.g., “not involved”) or when they lacked sufficient information (“I don't know”). Before scoring, all participants received the same operational definitions: Clarity referred to how easy the item was to understand, Relevance to how well it fit their context, and Usability to how feasible the idea would be to apply in practice. Definitions and response anchors were identical across roles; no role-specific examples were provided.

#### 4.4.1 Hypothetical fairness risk scenario used in the interview questionnaire

To situate interviews in a concrete fairness context, the questionnaire included the Hypothetical Fairness Risk Scenario, based on anonymized microdata from the *UNHCR Poland, Slovak Republic, Hungary, Czech Republic, Moldova, Romania, Bulgaria: Multi-Sector Needs Assessment (MSNA) - 2023* (SocioFactor et al., 2023). HealthEmove's privacy-preserving data architecture did not provide the project team with access to the contents of users' EPHRs. The scenario was deliberately simplified to provide a common, readily understandable reference point for participants with different levels of technical and fairness expertise. It served as a hypothetical elicitation exercise rather than an ecologically faithful representation of the envisioned HealthEmove feature. Accuracy was used solely as an accessible numerical cue for reflection and was not treated as a fairness metric or evidence of a fairness disparity (Matos et al., 2026). The envisioned feature is not intended for clinical diagnosis or treatment decision-making, and the scenario was not designed as a clinical decision-support or medical-device performance evaluation.

As presented in the questionnaire, the scenario concerned a hypothetical HealthEmove AI feature that would aim to predict whether an uploaded medical document contains a chronic health condition. To construct this scenario, we trained a binary logistic regression model on anonymized UNHCR MSNA 2023 data using Python v3.12.13. The model predicted self-reported chronic condition status using recent health problems, access to care, reported health access barriers, and age. Because the proxy model used structured survey variables, it did not reproduce the image-based information-extraction process or operational context of the anticipated feature. The scenario analysis comprised 5,274 adults with non-missing outcome and predictor values. For the subgroup comparison, women aged 60 years and older were compared with all other adults. Data were split into training and test sets using a 70/30 partition with stratification on the outcome and a fixed random seed. The administered questionnaire reported accuracy as approximately 21 percentage points higher for women aged 60 years and older than for other adults. It presented this difference as a hypothetical monitoring concern for participants to assess. The proxy model was not used to evaluate a deployed HealthEmove system, to establish fairness outcomes, or to test mitigation strategies. The focal subgroup combined sex and age; however, this one-versus-rest comparison was not a comprehensive intersectional audit, and further disaggregation would be required in any operational system evaluation. A *post-hoc* technical characterization of the proxy model and redacted analysis code are provided in Supplementary material H. Requests for further code details may be directed to the corresponding author, subject to the UNHCR Microdata Library Terms of Use and applicable access restrictions.^5^

Following the scenario description, participants provided 1–5 scores assessing perceived seriousness of the fairness concern, confidence in the project's ability to identify and interpret it, importance of taking action, need for cross-role coordination, relevance for equitable support, and potential effects on user trust, with optional explanatory comments. UNHCR does not warrant in any way the accuracy of the data or information reproduced from the Microdata Library and may not be held liable for any loss caused by reliance on the accuracy or reliability thereof. The analyses, interpretations, and conclusions reported in this study are solely those of the authors.

### 4.5 Data preparation and analysis

Oral interviews were transcribed using Amberscript or Microsoft Teams, while written responses were entered directly by participants. Transcripts were cleaned for readability only; meaning was not altered. Hesitations, repetitions, backtracking, clarifications, and tangential remarks were retained or marked where relevant. Written responses were retained verbatim, without paraphrasing. All responses were organized in a pseudonymized long-format spreadsheet, with each row representing one participant–item pair. Variables included pseudonymized participant identifiers, role, questionnaire item identifiers, domain classification, score type, and score value. Likert-scale responses were stored for all scored items, including CRU scores for F2A items 1–22 and single-score items from the Framework Assessment, Scenario-Based Evaluation, and Interview Questionnaire Design sections.

Qualitative coding was conducted in ATLAS.ti (macOS version 25.0), using one document per participant. Only responses to F2A items 1–22 were segmented and coded. Qualitative analysis followed a framework-guided, item-level qualitative content analysis approach that combined deductive and inductive coding. Parent codes were deductively derived from the predefined F2A prompt structure, while sub-codes were developed inductively from interview content to capture recurring patterns within each parent code. Coding used a fixed set of 22 parent codes corresponding to the F2A domains: five *What*, 11 *How*, and six *Why* codes. The primary researcher conducted both coding stages; the resulting coding was subsequently reviewed by a secondary coder. During the deductive coding stage, responses were segmented into quotations and coded with the corresponding parent code; segments were created only when a new analytic idea relevant to the item was introduced. A quotation was defined as a segment of text expressing one coherent idea relevant to a single F2A item. During the inductive refinement stage, quotations were reviewed across participants within each parent code, and subcodes were created when recurrent patterns or analytically important distinctions required clarification. The definitions of the 22 parent codes remained fixed, and quotations were not merged, split, resized, or deleted. Code saturation was not used as an analytic stopping criterion. The case-bounded, role-stratified sample and 22-prompt coding frame were specified before analysis, and recruitment was not extended in response to emerging subcodes. The analysis therefore sought framework-guided coverage and cross-role variation rather than exhaustive saturation.

Analytic memos supported interpretation. Item-level code memos summarized recurring patterns, distinctions, tensions, and absences within each F2A code. Domain memos synthesized patterns across the *What, How*, and *Why* dimensions. Document memos captured role context and notable or deviant responses, and a project memo recorded analytic decisions and code boundary clarifications. These memos served as the primary link between coded data and reported findings.

Quantitative and qualitative data were analyzed separately using methods appropriate to each data type. The spreadsheet was used for descriptive quantitative analysis and ATLAS.ti for qualitative analysis. Clarity, relevance, and usability were each rated on a 1–5 Likert scale (1 = very low, 2 = low, 3 = moderate, 4 = high, and 5 = very high) and analyzed descriptively at the item level. Means and SDs were retained as descriptive summaries only; given the small sample and single-item ratings, differences between items were not interpreted as reliable. Any CRU score of 1 was pre-specified to trigger item-level review and possible prompt revision. Because individual roles were represented by one participant and broad role groupings were small, no cross-role disagreement threshold was pre-specified. No inferential statistics or statistical comparisons across roles were performed. As a sensitivity analysis, aggregate CRU means were recalculated after excluding the three co-author participants. CRU scores were treated as self-reported, item-level formative indicators of perceived interpretability and practical fit. Internal consistency was not estimated because the 22 prompts represent distinct F2A constructs and the CRU ratings were not designed as interchangeable indicators of a single latent scale. Ratings were collected once, so temporal stability was not assessed. Cross-item variation was examined descriptively through the item-level distributions reported in Supplementary material I. Framework Assessment, Scenario-Based Evaluation, and Interview Questionnaire Design items were also analyzed descriptively in the spreadsheet.

To support analytic credibility, a secondary coder reviewed the complete read-only ATLAS.ti project after primary coding. The review covered all 241 open-ended responses and assessed segment inclusion and fit, parent-code and subcode assignments, coding level, and analytic scope. The coder recorded 84 question-level observations concerning 83 responses: 27 coding disagreements requiring resolution (10 concerning code assignment or interpretation, 10 coding level, and seven segment inclusion or analytic scope) and 57 clarifications, supplementary coding suggestions, or confirmations of existing coding. All 27 disagreements were resolved through discussion and consensus; final coding decisions remained with the primary researcher. In the final coding, 12 resolutions aligned with the secondary coder's position, seven reflected partial or alternative resolutions, and eight retained the primary researcher's position. The secondary coder did not independently recode transcripts. Because the review did not yield paired independent coding decisions at the segment-code unit, neither percentage agreement nor an inter-coder reliability coefficient was calculated. The secondary coder, a co-author, was not involved in developing F2A, HealthEmove, data collection, or primary coding. Participants did not review final findings. Trustworthiness was supported by an audit trail of coded transcripts, datasets, memos, and the disagreement log, alongside secondary-coder review. The primary researcher, an algorithmic fairness researcher, participated in Digital Health for All consortium meetings for this study and conducted all primary coding. This contextual familiarity may have influenced interpretation; analytic and reflexive memos documented coding decisions and boundary clarifications.

The Supplementary materials are organized as follows: (A) analysis and coding protocol, final codebook, and de-identified table documenting the resolution and resulting change for each coding disagreement; (B) data protection and ethics documentation checklist; (C) interview questionnaire; (D) interview protocol; (E) RACI matrix; (F) role-specific F2A guidance artifact; (G) COREQ checklist (Tong et al., 2007); (H) technical characterization of the hypothetical fairness risk scenario and redacted analysis code; and (I) item-level CRU summary statistics and score frequencies.

### 4.6 Stakeholder responsibility mapping and role-specific instantiation

A structured RACI analysis was conducted to map fairness-related responsibilities, decision authority, and knowledge dependencies within the HealthEmove ecosystem. The mapping was based on project documentation and interview transcripts to identify how fairness-related tasks were currently distributed, enacted, or expected across roles. The matrix-completion procedure instructed contributors to document disagreement or uncertainty in the Notes column rather than force an assignment; remaining ambiguities were addressed through iterative consultation with project leadership. The RACI matrix was constructed at the level of project roles rather than individuals. For each of the 22 F2A tasks, roles were assigned a single RACI designation. Exactly one role was designated as Accountable per task, while multiple roles could be designated as Responsible, Consulted, or Informed. Roles not involved in a given task were left unassigned. This design enabled comparability across tasks and ensured traceability between F2A items, empirical data, and responsibility assignments. The RACI mapping served two purposes: to render implicit responsibility structures explicit and to support role-specific instantiation of F2A.

Role-specific instantiation was conducted in two steps, followed by formative evaluation using the same CRU criteria applied to the F2A prompts. The study examined the instantiation process as a bounded proof of concept rather than comparing artifacts across roles; therefore, one role was selected for full instantiation. The Junior Researcher (JR) was selected because the role was represented in both the interview data and RACI mapping and was assigned Responsible status for multiple F2A tasks whose enactment depended on coordination with other roles. In the RACI mapping, the JR role was not assigned Accountable status for any F2A task. A closely collaborating role was included to provide a perspective beyond the target role holder's self-assessment. First, interview responses were analyzed to identify role-specific knowledge and practice gaps related to the operationalization of algorithmic fairness. These gaps included difficulties in recognizing fairness-relevant situations, limited access to actionable guidance, and reliance on informal processes. Based on these patterns, a recommendation page was developed consisting of concise, role-facing working instructions. Recommendations were derived from recurring interview patterns and formulated to support routine awareness, documentation, and escalation of fairness-related issues during project work.

Second, the checklist page was derived directly from the RACI analysis. F2A tasks assigned to the JR as Responsible were selected and translated into checklist items. Each item preserved the underlying F2A task while being operationalized into context-specific triggers (i.e., situations in which the task becomes relevant), required actions, and purposes. Required actions were structured around documenting observations, decisions, and follow-up steps in a shared “Fairness Knowledge Document” introduced as a proposed documentation template to support traceability and coordination in practice. Consulted and Informed roles were retained as reference columns, preserving the coordination structure defined in the RACI matrix. The structure of the checklist (trigger, required action, purpose, consult, and inform) was informed by existing reporting and assessment frameworks and implemented to support consistent documentation and role coordination in practice. This procedure ensured traceability from F2A prompts to checklist items while aligning tasks with mapped role responsibilities and observed knowledge gaps. The resulting artifact consisted of two complementary components: an interview-informed recommendation page and a RACI-derived checklist page. The instantiated instrument underwent preliminary formative assessment using the CRU criteria. The selected target role holder and the secondary role completed 1–5 CRU ratings for the recommendations, checklist items, and document overall, accompanied by qualitative feedback. Descriptive reporting comprised evaluator-specific scores, means, and sample SDs; SDs were not calculated where only one rating was available.

### 4.7 Ethical considerations and potential biases

The study involved adult professional stakeholders only and did not involve patients, clinical intervention, patient-level data, identifiable health records, sensitive or special-category personal data, medical or invasive procedures, or vulnerable populations as research participants. All participants received an information sheet and provided informed consent before participation. Participation was voluntary. Participants were informed that they could skip any question or stop participation at any time without explanation or consequence, and no compensation was provided. Data handling followed University of Amsterdam data protection guidance and GDPR principles, including data minimization, restricted access, pseudonymization prior to analysis, and measures to reduce attribution risk in reporting. The HealthEmove project name is reported with permission; participants were also offered the option of anonymizing the project name in reporting. Direct identifiers, such as participant names, were not retained in the analysis files. Analysis files used pseudonymized participant identifiers and role-related information. Interview recordings, where applicable, were stored separately from transcripts, written responses, and extracted scores and will be deleted after project completion in accordance with the participant information sheet. Transcripts, written responses, and extracted scores were analyzed using pseudonymized participant identifiers and stored in a restricted-access research environment. UNHCR Microdata Library data used for the fairness scenario in the interview questionnaire were accessed and analyzed in accordance with the applicable Terms of Use and solely for the approved scientific research purposes of this study. The participant-facing information sheet, consent section, and interview questionnaire are provided in Supplementary material C, and additional documentation of the consent procedure, data protection checklist, and data management plan is provided in Supplementary material B.

Because the study was conducted within a small project team, some responses could be contextually recognizable to people familiar with HealthEmove. To reduce attribution risk, the results are reported using role groupings and without linking responses to specific individuals. The primary researcher participated in Digital Health for All consortium meetings resulting in familiarity with the project context, which supported interpretation but introduced potential bias. This was addressed through systematic coding procedures, analytic and reflexive memos, secondary-coder review, decision logging, and discussion of disagreements, as documented in Supplementary material A. These safeguards helped increase transparency. Co-author participant roles are disclosed in the Conflict of Interest statement.

## 5 Results

This section reports the empirical findings of the study. The results are presented in five parts: (1) participant overview and response characteristics; (2) qualitative findings on fairness knowledge translation; (3) prompt-level ratings of Clarity, Relevance, and Usability; (4) role-specific artifact instantiation through stakeholder responsibility mapping; and (5) synthesis of main findings.

### 5.1 Participant overview and response characteristics

The analysis draws on responses from 11 participants, comprising four in primarily technical roles and seven in primarily managerial or governance-oriented roles within the HealthEmove project. Data were collected using a structured interview questionnaire comprising three main sections and an appendix: (1) 22 F2A prompt items, each with associated CRU scores; (2) 6 framework assessment items; (3) 6 scenario-based evaluation items; and (4) 7 interview questionnaire design items. The dataset included 241 open-ended responses to the 22 F2A prompts, from 242 possible participant-prompt combinations (11 participants × 22 prompts), together with the available CRU ratings. Across the questionnaire, there were 451 possible participant-item combinations (11 participants × 41 items). Item-level completeness was high, with occasional “I don't know” responses retained and interpreted as indicators of role-specific knowledge boundaries rather than missing data. To reduce re-identification risk in a role-identifiable project setting, participant identifiers and exact role labels are generally not reported in the Results. Where analytically relevant, responses are reported using broad role groupings distinguishing primarily technical roles from primarily managerial or governance-oriented roles; exact role labels are used only where required for the role-specific artifact instantiation.

### 5.2 Qualitative findings on fairness knowledge translation

Open-ended responses to the 22 F2A prompts are reported by item within the framework's *What, How*, and *Why* dimensions. The findings include accounts of existing and aspirational practices unless otherwise noted, and item headings use condensed prompt formulations.

#### 5.2.1 F2A: what is the current state of the algorithmic fairness knowledge gap?

Fairness knowledge was unevenly distributed across roles and only partly operationalized. Participants commonly grounded their awareness in ethical concerns, prior exposure, or risks such as bias and data quality, while some named concrete methods, benchmarks, testing, or documentation practices. One participant captured the gap between recognizing fairness and knowing how to apply it:

“When we speak of data systems and making them algorithmically fair, [I have] very little knowledge.”

##### 5.2.1.1 Knowledge availability: what fairness knowledge or guidance exists?

Participants distinguished recognizing fairness from knowing how to act on it. Many, particularly in managerial or governance-oriented roles, described their knowledge as partial, high-level, or developing. Several located relevant knowledge among technical experts, researchers, regulators, or external advisors but were uncertain what it entailed or how it applied to HealthEmove. Technical participants more often named concrete fairness concepts and practices, including model limitations, testing, and documentation, whereas managerial or governance-oriented participants more often located knowledge elsewhere or described it as developing.

##### 5.2.1.2 Responsibility holders: who has the main fairness responsibility or expertise?

Fairness-related knowledge was distributed rather than concentrated. Technical roles were most often associated with model-level expertise; project leadership, product ownership, and governance roles with accountability, prioritization, and final decisions. Some participants argued that system builders should not define fairness alone and referred to advisory or independent oversight structures that should exist but were not yet in place. Intended users were rarely directly involved in defining fairness-related solutions, although end-user and living-lab input was more often cited as contextual knowledge.

##### 5.2.1.3 Knowledge sources: where does fairness knowledge or advice come from?

Participants identified internal technical experts, project leadership, academic literature, legal advisors, regulatory frameworks, AI providers, external experts, and living-lab or end-user input as fairness knowledge sources. Internal sources were described as practical and discussion-based, and legal, regulatory, and external sources as boundary-setting or advisory. Few described a shared consolidation mechanism, although several indicated its need.

##### 5.2.1.4 Knowledge access: how do team members access fairness information or support?

Access to fairness knowledge was usually informal and situational, through recurring meetings, conversations with knowledgeable colleagues, academic reading, online searches, and *ad hoc* consultation. Many sought more stable mechanisms, including a shared knowledge base, curated documentation, examples, checklists, dashboards, or designated contacts. One participant proposed:

“Yeah, I think it could be made more readily available by having a a shared database on good sources and learnings in that sense like a know-how document that can be spread through the team as well. Like a knowledge package.”

##### 5.2.1.5 Knowledge effects: how is the effect of fairness efforts assessed and reported?

Participants generally considered fairness knowledge relevant to model selection, feature design, exclusions, prompting, user protection, and anticipated monitoring. Its effects, however, were indirect, preparatory, or conditional rather than embedded in formal decision criteria: It informed discussion, raised design concerns, or motivated planned adjustments rather than guiding a stable process.

#### 5.2.2 F2A: how is algorithmic fairness knowledge transferred?

Fairness knowledge transfer was described as informal, situated, and practice-based, occurring through incremental discussion shaped by data availability, context, and project maturity. Some participants mentioned structured mechanisms developed for other purposes, such as ticket systems.

##### 5.2.2.1 Inquiry process: how are new fairness lessons generated or captured?

Fairness questions usually arose through individual initiative, recurring discussion, external prompting, or specific design concerns rather than shared lifecycle procedures. Some described more structured mechanisms, including logging user concerns, post-deployment monitoring, audits, vigilance, and risk management, as emerging or still developing rather than established practice. As one participant summarized:

“So I guess [HealthEmove] captures it now through the individual members getting up to speed on certain concepts and then doing something with it. But it's not that institutionalized I would say. It's more informal.”

##### 5.2.2.2 Knowledge synthesis: how is fairness knowledge synthesized from sources?

Fairness knowledge was synthesized through team discussion, exchanges with technical experts, or consultation among partners. Several participants sought clearer coordination through designated ownership, shared documentation, or a maintained knowledge base but described these mechanisms as incomplete, emerging, or absent.

##### 5.2.2.3 Fairness tools: what mechanisms support the use of fairness knowledge?

Participants sought practical supports, including benchmarks, checklists, templates, guidelines, dashboards, testing procedures, examples, and shared documentation. Several considered checklist-based support potentially useful for daily work. Existing supports were partial, general, or under development rather than dedicated fairness tools embedded in workflow.

##### 5.2.2.4 Needs identification: how are fairness issues identified in practice?

Fairness issues were described as becoming visible through testing, user or living-lab input, known model limitations, practical design concerns, or reflection on possible harms. Several participants also indicated that some fairness issues are difficult to identify in advance, especially in a project serving highly diverse users and still developing its AI-enabled features.

##### 5.2.2.5 Knowledge selection and adaptation: how is fairness knowledge selected and adapted to the project setting?

Participants prioritized feasibility, contextual fit, and practical usability over formal method comparison when selecting fairness approaches, describing adaptation as tied to HealthEmove's user population, including vulnerability, linguistic diversity, mobility, privacy sensitivity, and healthcare relevance, and to trade-offs with privacy, usability, safety, and feasibility. Some indicated that clearer benchmarks, standards, or ready-to-use process artifacts would facilitate selection.

##### 5.2.2.6 Barrier assessment: what barriers make fairness goals difficult to act on?

Reported barriers included limited knowledge, lack of clear ownership, dependence on senior or external actors, limited time or capacity, absence of dedicated structures, data limitations, lack of access to model internals, privacy constraints, and competing project priorities. Several participants also described fairness as one priority among others rather than as a consistently dominant criterion.

##### 5.2.2.7 Intervention implementation: what concrete actions are taken or could be taken?

Participants mentioned testing with diverse users, prompt design, output monitoring, human-in-the-loop checks, awareness-raising, documentation, user feedback loops, and structured reviews of possible fairness risks. These actions varied across roles and were often partial, provisional, or under development.

##### 5.2.2.8 Use monitoring, outcome evaluation, and use sustainment: how are fairness issues and practices tracked, reviewed, and sustained over time?

Participants considered monitoring, evaluation, and sustainment necessary but inconsistently developed. Suggested mechanisms included dashboards, logs, complaints or feedback channels, recurring meetings, testing flows, mock data, audits, checkpoints, documentation, handover, and maintained knowledge bases or wiki-like resources. Sustainment was linked to documentation, role continuity, and embedding fairness in routines rather than individual initiative. One participant described incorporating complaints into recurring review:

“Yeah, like a log or something. Your customer care could, of course, highlight complaints about this. And you can make sure that that comes back in your weekly meetings, for example, that you always discuss items that have popped up on a list like that.”

#### 5.2.3 F2A: why does the algorithmic fairness knowledge gap persist?

Participants considered fairness relevant but identified barriers to routine use: competing priorities, limited practical supports, uneven incentives, and differing understandings across roles and organizations.

##### 5.2.3.1 Fairness framing: is fairness mainly technical, or also ethical and social?

Participants most often framed algorithmic fairness as an ethical and social responsibility, while also recognizing a technical dimension in some accounts. Fairness was described in relation to users, inclusion, trust, accountability, or broader business or governance responsibility. Some participants explicitly linked technical design choices to social consequences, while others emphasized that fairness should be treated as a core organizational or product responsibility.

##### 5.2.3.2 Action gap: is fairness mostly discussed, or also acted upon?

Participants described a gap between discussing fairness and embedding it in routine action. In some accounts, fairness remained conceptual; in others, it was regularly discussed and linked to concrete action but lacked stable procedures, benchmarks, or follow-through. Reasons included absent practical methods, uncertain next steps, and uncertainty about whether fairness was a visible project priority. As one participant stated:

“Behavior doesn't change unless there is a value for business.”

This view was not universal, but several responses similarly linked action to visible value, explicit prioritization, or stronger governance pressure.

##### 5.2.3.3 Routine reporting: is fairness work about reporting only, or learning and improvement?

Participants considered reporting most useful when supporting learning and improvement rather than isolated box-checking, linking it to review, evaluation of changes, and iterative adjustment. Some technical or governance-oriented accounts also valued documentation, auditability, and traceability for compliance, quality control, or formal oversight. Reporting varied across participants but was more often considered meaningful when connected to follow-up or accountability.

##### 5.2.3.4 Reputational concerns: do performance or reputational concerns discourage openness?

Reputational concerns were described variably. Some participants treated them as hypothetical or not currently dominant in the project, while others linked fairness disclosure to additional work, slower delivery, internal friction, or reluctance to surface difficult issues openly. In these accounts, the concern was often less about reputation in the abstract than about the practical costs of disclosure, remediation, or delay.

##### 5.2.3.5 Metric dominance: are accuracy or efficiency sometimes prioritized over fairness?

Participants said fairness should be balanced with other goals, although these goals varied. Accuracy, efficiency, speed, simplicity, or delivery requirements could outweigh fairness concerns in practice, especially in early development or when a system was seen as improving a poor status quo. Others argued that fairness and performance should be considered jointly rather than directly traded off. Fairness was rarely rejected but was not always the dominant decision criterion.

##### 5.2.3.6 Disciplinary divides: do disciplinary divides affect fairness adoption?

Participants did not uniformly consider multidisciplinary collaboration a barrier. Several valued collaboration among legal, technical, public health, and user-facing roles for broadening fairness-related perspectives. Participants also cited differences in technical understanding, priorities, communication, and dependence on actors outside the core team. Early alignment, open communication, and clearer shared priorities could reduce these difficulties. One participant described the difficulty of translating organizational expectations into practice:

“Yeah, I think the the further removed the organizations are from the actual day-to-day work, the more they want fairness and the less they understand what it may be. So it's like a concept that is very difficult to turn into practice.”

#### 5.2.4 Cross-role similarities and differences

HealthEmove and DFD participants considered fairness important, particularly for HealthEmove's vulnerable users, and described responsibility for fairness knowledge as distributed among technical, leadership, product, governance, legal, and external actors.

HealthEmove participants emphasized awareness-building, discussion, and clearer guidance through meetings, examples, and shared reflection to move from discussion to action, whereas DFD participants more often described procedural, technical, or governance-oriented mechanisms, including testing, dashboards, monitoring, risk management, documentation, checklists, vigilance, post-market surveillance, governance frameworks, and business requirements. Although some mechanisms were incomplete or prospective, these accounts specified workflows, controls, procedures, or oversight structures for operationalization.

Both groups cited competing priorities, limited time or capacity, dependence on other actors, and tensions between fairness and privacy, usability, performance, delivery, or broader product value. Reputational concerns were less consistent, but several participants said fairness was harder to prioritize when it created additional work, slowed progress, or lacked clear ownership. Confidence that fairness issues would be identified was mixed, especially without firmly established monitoring or review structures.

### 5.3 Prompt-level ratings of clarity, relevance, and usability

Across the F2A framework, CRU scores were generally positive, with most item means at or above 4 on the 5-point scale (Table 1). Across all 22 items, mean scores were 4.4 for Clarity, 4.4 for Relevance, and 4.2 for Usability. Of the 722 valid ratings, 616 (85.3%) were 4 or 5, 80 (11.1%) were 3, and 26 (3.6%) were 2. No score of 1 occurred. Consequently, the pre-specified threshold was not triggered, and no prompt was revised on this basis. All item-level medians were 4 or 5. Supplementary material I reports item-level medians, IQRs, and score frequencies.

**Table 1:** Clarity, Relevance, and Usability (CRU) scores for the 22 F2A framework items across the *What, How*, and *Why* dimensions.

Dimension	Item	F2A question (condensed version)	Clarity	Relevance	Usability
What	Knowledge availability	What fairness knowledge or guidance exists?	3.6 (1.1)	4.4 (0.7)	4.1 (0.9)
	Responsibility holders	Who has the main fairness responsibility or expertise?	**4.7** (0.5)	4.4 (0.9)	**4.5** (0.7)
	Knowledge sources	Where does fairness knowledge or advice come from?	4.6 (0.5)	**4.5** (0.5)	4.0 (0.8)
	Knowledge access	How do team members access fairness information or support?	4.1 (1.0)	**4.5** (0.5)	4.2 (0.8)
	Knowledge effects	How is the effect of fairness efforts assessed and reported?	4.4 (0.7)	**4.5** (0.5)	4.4 (0.7)
How	Inquiry process	How are new fairness lessons generated or captured?	4.4 (0.9)	**4.6** (0.7)	4.2 (0.9)
	Knowledge synthesis	How is fairness knowledge synthesized from sources?	4.5 (0.5)	4.4 (1.0)	**4.5** (1.0)
	Fairness tools	What mechanisms support the use of fairness knowledge?	4.5 (0.8)	4.4 (0.9)	4.3 (1.0)
	Needs identification	How are fairness issues identified in practice?	4.0 (1.1)	4.5 (0.7)	4.1 (0.8)
	Knowledge selection	How is fairness knowledge selected for use?	3.9 (0.8)	4.2 (1.1)	4.0 (1.0)
	Knowledge adaptation	How is fairness adapted to the project setting?	4.1 (0.8)	4.4 (0.5)	4.2 (0.8)
	Barrier assessment	What barriers make fairness goals difficult to act on?	4.5 (0.5)	4.5 (0.7)	4.2 (1.1)
	Intervention implementation	What concrete actions are taken or could be taken?	4.6 (0.7)	4.5 (0.7)	4.2 (0.9)
	Use monitoring	How is fairness use monitored over time?	4.4 (0.8)	4.4 (0.8)	4.2 (1.0)
	Outcome evaluation	How is learning from past fairness experience supported?	4.5 (0.5)	4.5 (0.7)	4.3 (0.6)
	Use sustainment	How is fairness use sustained in practice?	**4.7** (0.5)	4.5 (0.7)	4.4 (1.0)
Why	Fairness framing	Is fairness mainly technical, or also ethical and social?	**4.6** (0.5)	4.1 (1.0)	**4.5** (0.7)
	Action gap	Is fairness mostly discussed, or also acted upon?	**4.6** (0.5)	**4.5** (0.7)	4.0 (1.1)
	Routine reporting	Is fairness work about reporting, or learning and improvement?	4.1 (0.9)	4.2 (0.8)	4.3 (0.7)
	Reputational concerns	Do performance or reputational concerns discourage openness?	**4.6** (0.5)	4.3 (1.0)	4.0 (1.0)
	Metric dominance	Are accuracy or efficiency sometimes prioritized over fairness?	4.4 (0.7)	3.9 (1.0)	3.9 (0.8)
	Disciplinary divides	Do disciplinary divides affect fairness adoption?	4.4 (0.8)	4.2 (0.9)	3.9 (0.9)

Values are mean (SD) based on available responses. Higher scores indicate that participants perceived an item as easier to understand, more relevant to their role and context, or more feasible to apply in practice. Scores were measured on a 5-point Likert scale (1 = very low, 5 = very high). The largest observed descriptive mean(s) within each framework dimension are shown in bold for visual reference; differences between items are not interpreted as reliable. For all items, *n* = 11 except Relevance for *Knowledge Adaptation* and all three CRU dimensions for *Routine Reporting*, where *n* = 10.

Three of the 11 CRU respondents were co-authors; no participant was involved in developing F2A or the materials used for its formative evaluation. Excluding the three co-author respondents (*n* = 8) reduced mean Clarity from 4.4 to 4.3, Relevance from 4.4 to 4.2, Usability from 4.2 to 4.0, and CRU overall from 4.3 to 4.2.

Overall, the self-reported formative ratings were generally favorable. However, the small sample, single-item ordinal ratings, and concentration of responses at the upper end of the scale preclude reliable comparisons between prompts.

#### 5.3.1 Overall framework assessment

As shown in Table 2, Framework Assessment scores were more positive for structure and coverage than for immediate practical application. Participants generally described the framework as broad in scope, useful for surfacing fairness-related issues, and relatively strong in coverage. Comments on practical use more often pointed to the need for clearer implementation pathways, examples, and role-specific operationalization. Lower scores on Practical Usefulness and especially Practical Feasibility were commonly linked to workload, competing priorities, abstraction, and the absence of sufficiently concrete guidance. In response to the question *How feasible would it be for your team to apply F2A in practice?*, one participant emphasized that practical uptake would depend on whether the framework could be made more directly operational:

**Table 2:** Overall framework assessment, scenario-based evaluation, and interview questionnaire design feedback scores.

Overall framework assessment		Scenario-based evaluation		Interview questionnaire design	
Item	Score	Item	Score	Item	Score
Structure clarity	3.8 (0.6)	Risk seriousness	3.9 (1.0)	Questions clarity	4.0 (0.0)
Structure fit	3.5 (0.8)	Team confidence	3.3 (1.3)	Instructions ease	4.2 (0.4)
Completeness framework	3.8 (0.8)	Take action	4.2 (0.8)	Length & workload	3.8 (0.7)
Completeness coverage	**3.9** (0.9)	Requires coordination	**4.4** (0.8)	Realistic & answerable	3.7 (0.5)
Practical usefulness	3.5 (1.1)	Ensure equity	4.1 (1.0)	Scale intuition	3.7 (0.9)
Practical feasibility	3.1 (0.8)	Affects trust	4.1 (1.0)	Assessment relevance	3.9 (0.3)
				Fields function	**4.3** (0.5)

Values are mean (SD) based on available responses. Scores were measured on a 5-point Likert scale (1 = very low, 5 = very high). Highest mean score(s) within each section are shown in bold. For Framework Assessment and Scenario-Based Evaluation, all items are based on *n* = 11. For Interview Questionnaire Design, Questions Clarity and Instructions Ease are based on *n* = 10, and the remaining items are based on *n* = 9.

“And it should be SMART. It should be actionable, measurable, that type of thing.”

#### 5.3.2 Scenario-based evaluation on a hypothetical fairness risk

As shown in Table 2, Scenario-Based Evaluation scores were highest for action and coordination, indicating that participants generally viewed the hypothetical fairness risk as requiring collective response rather than isolated individual action. Equity and trust implications were also scored relatively highly. By contrast, Team Confidence was lower and more variable, with comments pointing to uncertainty about whether such issues would be identified in practice. In response to the question *How confident are you in the team identifying and interpreting this fairness issue?*, one participant linked lower confidence directly to the absence of monitoring:

“I don't think the monitoring will be done. So, then [the fairness issue] won't get noticed.”

These scores are interpreted as responses to the hypothetical risk as presented, rather than as participant validation of the proxy model, its metrics, or its ecological validity.

#### 5.3.3 Interview questionnaire design feedback

As shown in Table 2, Interview Questionnaire Design scores were positive overall, with the strongest support for response format, clarity, and ease of use. Participants generally described the questionnaire as clear, manageable, and relevant to the project context. Lower scores clustered around answerability and scale intuition, and comments indicated that some questions required additional explanation or reflection, particularly where concepts were more abstract. In response to the question *How clear did the questions feel overall?*, one participant noted:

“Sometimes explanation was needed.”

Additional comments referred to questionnaire length and the level of detail requested for certain items. Reported completion time averaged 44.40 min (*SD* = 16.23).

Overall, these formative assessment results complement the item-level F2A findings. Within each component, ratings were comparatively higher for framework structure and coverage, the importance of action and need for coordination in response to the hypothetical risk, and questionnaire clarity and fillable-field functionality. Ratings were comparatively lower for the framework's immediate feasibility in practice, team confidence in identifying and interpreting the hypothetical risk, and the realism and answerability of the open-ended items.

### 5.4 Role-specific artifact instantiation through stakeholder responsibility mapping

To translate F2A into role-specific guidance, we instantiated the framework for the JR role. The artifact consisted of a two-page, role-specific document combining (1) concise recommendations for the day-to-day work of the JR and (2) a structured checklist derived from selected F2A tasks and the RACI mapping, organized around triggers, required actions, purposes, and consult/inform roles. This checklist-based format aligned with earlier qualitative findings, in which participants identified practical supports such as checklists, examples, templates, and shared documentation as potentially useful for day-to-day fairness work. The checklist translated mapped F2A items into context-specific tasks while linking actions to documentation in a shared *Fairness Knowledge Document*. The RACI task matrix used for responsibility mapping is provided in Supplementary material E, and the instantiated artifact is provided in Supplementary material F; the completed role-assignment matrix can be made available upon reasonable request, subject to project confidentiality and attribution-risk constraints.

The instrument underwent preliminary formative evaluation by two of the 11 participants: the JR role itself and a closely collaborating Research Assistant (RA). The assessment covered component-level CRU ratings, overall document assessment, and open-ended feedback. Evaluator-specific scores, item-level means, and sample SDs are reported in Table 3.

**Table 3:** Preliminary formative evaluation scores for the role-specific guidance artifact.

Item	Clarity	Relevance	Usability
Recommendation page			
Instructions and overall explanation	5.0 (NA); 5/–	4.0 (NA); 4/–	5.0 (NA); 5/–
Fairness knowledge document	5.0 (0.0); 5/5	5.0 (0.0); 5/5	5.0 (0.0); 5/5
Recording situations	5.0 (0.0); 5/5	4.5 (0.7); 5/4	4.0 (1.4); 5/3
Trigger questions	5.0 (0.0); 5/5	5.0 (0.0); 5/5	4.5 (0.7); 5/4
Escalation guidance	5.0 (0.0); 5/5	4.0 (0.0); 4/4	3.5 (0.7); 4/3
Handover and continuity	5.0 (0.0); 5/5	5.0 (0.0); 5/5	5.0 (0.0); 5/5
Sources for further guidance	5.0 (NA); 5/–	4.0 (NA); 4/–	4.0 (NA); 4/–
Checklist page			
Checklist instructions	5.0 (0.0); 5/5	5.0 (0.0); 5/5	5.0 (0.0); 5/5
F2A-1: knowledge availability	5.0 (0.0); 5/5	5.0 (0.0); 5/5	5.0 (0.0); 5/5
F2A-6: inquiry process	4.0 (1.4); 5/3	4.5 (0.7); 5/4	4.5 (0.7); 5/4
F2A-7: knowledge synthesis	5.0 (0.0); 5/5	5.0 (0.0); 5/5	5.0 (0.0); 5/5
F2A-12: barrier assessment	4.0 (1.4); 5/3	4.5 (0.7); 5/4	4.5 (0.7); 5/4
F2A-15: outcome evaluation	5.0 (0.0); 5/5	5.0 (0.0); 5/5	5.0 (0.0); 5/5
F2A-19: routine reporting	5.0 (0.0); 5/5	5.0 (0.0); 5/5	5.0 (0.0); 5/5
Roles and glossary	5.0 (0.0); 5/5	4.5 (0.7); 4/5	5.0 (0.0); 5/5
**Item**	**JR score**	**RA score**	**Mean (SD)**
Overall document assessment			
Overall clarity	5	5	5.0 (0.0)
Overall relevance	4	4	4.0 (0.0)
Overall usability	4	4	4.0 (0.0)
Completeness	4	5	4.5 (0.7)
Usable without additional guidance	4	3	3.5 (0.7)

Component values are mean (SD), followed after the semicolon by the evaluator-specific scores in Junior Researcher/Research Assistant (JR/RA) order. Scores were measured on a 5-point Likert scale (1 = very low, 5 = very high). A dash indicates that no rating was provided; NA indicates that the SD could not be calculated from one rating.

For the recommendations page, Clarity ratings were all 5, Relevance ratings ranged from 4 to 5, and Usability ratings ranged from 3 to 5. The *Fairness Knowledge Document* and Handover and Continuity received ratings of 5 from both respondents on all three dimensions. For Usability, Escalation Guidance received ratings of 3 and 4, Recording Situations received ratings of 3 and 5, and Sources for Further Guidance received one rating of 4. Given the two-person assessment, these ratings are reported descriptively and are not interpreted as reliable differences between components. One respondent reported that the recommendations were useful and that they had already begun acting on them. This single self-report was not treated as evidence of sustained use. Respondents also noted potential overlap between recording a fairness-related situation and recording a barrier requiring follow-up. One respondent suggested supplementing primary legal sources with explanatory commentary.

The checklist page was also rated positively. Checklist instructions received the maximum score (5) across all three dimensions from both respondents. Many checklist components received maximum or near-maximum ratings across respondents, particularly *Knowledge Availability, Knowledge Synthesis, Outcome Evaluation*, and *Routine Reporting*. Lower scores were concentrated on *Inquiry Process* and *Barrier Assessment*, which received ratings of 3 to 4 from one respondent. Comments linked these lower scores to conceptual overlap between the two categories rather than disagreement with their importance. Respondents also highlighted the usefulness of concrete examples in the trigger column and the inclusion of a *Purpose* column explaining why each action mattered. This feedback is consistent with the checklist format being perceived as a workable way of structuring role-specific fairness guidance in this project context.

At the document level, Clarity was rated 5 by both respondents, Relevance was rated 4 by both respondents, and Usability was rated 4 by both respondents. Completeness received ratings of 4 and 5. Ability to use the instrument without additional guidance received lower ratings of 3 and 4, indicating that some initial explanation or onboarding may improve independent uptake. Respondents also suggested practical refinements, including clearer expectations for documentation content and frequency, editable templates or flexible note-taking fields, and minor formatting changes to improve readability. One respondent cautioned that actual usability remained to be established through continued use and identified additional effort and unfamiliarity with the process as possible barriers to uptake.

Overall, this preliminary formative assessment records the perceptions of two internal respondents and does not establish independent usability or utility beyond those respondents. Their feedback identified possible needs for onboarding, clearer documentation expectations, and clarification of potentially overlapping checklist items.

### 5.5 Synthesis of main findings

Fairness knowledge was present in HealthEmove but asymmetrically distributed, only partly formalized, and often dependent on role-specific initiative. Participants generally viewed fairness as important, yet described practical uptake as constrained by competing priorities, unclear ownership, limited time or capacity, and the absence of dedicated tools, monitoring, and documentation. Internal, self-reported CRU ratings were concentrated at the upper end of the scale. Cross-role findings suggested that HealthEmove roles emphasized awareness and guidance, while DFD roles described more procedural and governance-oriented support. The proof-of-concept artifact for the Junior Researcher role received preliminary ratings from two internal respondents, indicating perceived practical fit within this case when connected to responsibility mapping.

## 6 Discussion

### 6.1 F2A as a knowledge translation instrument

Our study examined how the F2A framework can support the translation of fairness-related knowledge across roles in an applied digital health project. Across the qualitative and quantitative findings, participants generally viewed the F2A prompts as clear, relevant, and usable, yet fairness-related knowledge remained asymmetrically distributed and only partly embedded in shared routines. Interpretations varied with role, decision authority, proximity to implementation, and knowledge access. Within this formative case, F2A may therefore function as a structured means of examining whether fairness-related knowledge is available, interpretable, and usable in project work, rather than as a means of determining what fairness requires in a particular decision.

A core contribution is the proof-of-concept instantiation for the Junior Researcher role, which illustrates how responsibility mapping can convert selected F2A prompts into role-specific recommendations and a checklist linked to existing coordination structures. By making fairness expectations more concrete, the procedure addresses the difficulty of embedding fairness-related work into roles, routines, and decisions identified in this case.

The findings also indicate that interpretability alone does not ensure routine use. Although prompt-level CRU ratings were favorable, the mean completion time, comments on questionnaire length, and comparatively lower ratings for *Practical Feasibility* and *Realistic & Answerable* indicate possible respondent burden. Future refinement could therefore reduce completion demands and increase concreteness while preserving framework coverage. Lower practical fit was associated with unclear ownership, limited authority, absent tools or monitoring, and informal coordination. F2A is therefore best understood as a structuring device for examining how fairness knowledge is accessed, interpreted, and operationalized across roles, including where it exists or is missing and where action depends on organizational support, rather than prescribing decisions or technical solutions.

### 6.2 Role-sensitive translation and coordination

The results suggest that fairness knowledge translation is shaped by role position and decision authority. Participants did not simply differ in how much they knew about fairness; they also differed in how they located the problem, what counted as relevant action, and what kinds of support they considered necessary. Technical roles more often referred to data, system behavior, testing, monitoring, and feasibility constraints, whereas managerial, advisory, and governance-oriented roles more often emphasized responsibility, prioritization, accountability, oversight, and coordination. These differences are consistent with prior work suggesting that fairness-relevant decisions are distributed across actors rather than contained within a single technical role (Veale et al., 2018; Passi and Barocas, 2019). Accordingly, prompts and instantiated artifacts could emphasize testing and feasibility for technical roles and decision authority and oversight for managerial or governance roles.

This role sensitivity helps explain why fairness remained weakly embedded despite broad recognition of its importance: responsibilities, knowledge sources, and follow-up mechanisms were not stabilized, impeding coordination among reflection, authority, and action across roles. This aligns with work locating fairness implementation barriers in organizational routines and decision processes rather than awareness alone (Madaio et al., 2020; Rakova et al., 2021).

The RACI mapping is useful in this regard because it makes visible who is expected to act, who holds accountability, and where consultation or information flow is required. This addresses a recurring responsible AI problem in which responsibility can become displaced across distributed roles and organizational boundaries (Widder and Nafus, 2023). Rather than resolving fairness questions by itself, the mapping helps convert diffuse responsibility into a more explicit coordination structure. The instantiated artifact extends this structure by translating mapped responsibilities into task-level expectations, including triggers, required actions, purposes, and consult/inform relations. Connecting fairness-related knowledge to identifiable roles, concrete expectations, and recurring points of coordination may support its operationalization by strengthening the conditions under which concerns can be noticed, discussed, documented, and followed up, without prescribing a single fairness standard. This process orientation aligns with recent proposals to distribute fairness-related information-gathering and evaluation responsibilities between system providers and deployers rather than prescribe uniform outcome mandates (Raman et al., 2025). F2A complements this perspective at the project level by making role-specific responsibilities, dependencies, and information flows explicit while allowing their implementation to be adapted to local context. Assigning responsible and accountable roles to specific fairness tasks and documenting their dependencies may reduce the scope for fairness work to be implicitly transferred to another actor. Maintaining the mapping as roles and decision authority evolve may help guard against ethical offloading and preserve continuity in multi-institutional digital health projects.

### 6.3 Relation to existing frameworks

Existing fairness approaches specify what should be documented, evaluated, monitored, or governed through frameworks, audits, reporting standards, and governance guidance for transparency, review, and accountability (Mitchell et al., 2019; Raji et al., 2020; Collins et al., 2024). Evaluation research has examined the usability, feasibility, and workflow integration of such tools (Amershi et al., 2019; Madaio et al., 2020). However, these approaches often leave implicit whether project teams can make the required knowledge usable in their own settings.

Rather than evaluating tool adoption after implementation has begun, our study examines whether fairness-related knowledge is accessible and usable across roles before or while practices are being embedded. F2A could complement reporting and evaluation frameworks by organizing responsibilities, not producing the evidence or infrastructure they require. For TRIPOD+AI, Needs Identification and Knowledge Adaptation could define the target population, relevant health inequalities, and subgroup evaluation scope, while responsibility mapping could assign evidence production, review, and updating (Collins et al., 2024). Knowledge, tool, barrier, responsibility, and implementation prompts could support pre-deployment readiness checks; monitoring, evaluation, reporting, and sustainment prompts could define post-deployment indicators, review timing, escalation, remediation, and versioning (Wang et al., 2026; Bellibatlu et al., 2026; Weidinger et al., 2025). These conceptual links to downstream governance were not implemented or evaluated in HealthEmove and remain for future work.

Where existing literature often evaluates usability, adoption, or workflow integration, this study also contributes a procedure for instantiating F2A into role-specific artifacts. This matters because high-level prompts may not reach everyday project work without role-specific operationalization. F2A thus operates at the level of knowledge translation by structuring conditions for documentation, auditing, and review, rather than tool adoption alone.

### 6.4 Implications for digital health AI practice and policy

Several process-level implications arise from this single formative case and identify directions for further evaluation in comparable multi-stakeholder settings. First, fairness work in HealthEmove could not rely on high-level principles alone; general guidance may become more useful when translated into role-specific expectations reflecting decision authority, dependencies, and workflow constraints. Second, the case supports treating fairness as a coordination problem as much as a technical one. Lightweight responsibility mapping, shared documentation, and structured checkpoints may connect fairness reflection to project work without full procedural formalization at the outset. Third, fairness-related supports may need to remain adaptable and updateable as responsibilities, infrastructures, and system features change in evolving projects.

These implications are particularly relevant in digital health, where clinical, legal, organizational, and infrastructural constraints shape system design and harms may arise from technical choices or access inequalities. In HealthEmove, fairness depended not only on anticipated model behavior but also on stakeholder alignment about when to raise concerns, how to document decisions, and who would act. Checklists, examples, templates, and shared documentation may support such alignment when fairness work otherwise depends on informal initiative.

Potential implications for governance and policy also follow. Because fairness-related responsibilities are distributed, governance frameworks may need to specify how fairness work is coordinated, reviewed, and monitored across roles, not only state abstract requirements. This includes clearer accountability, documentation enabling traceability and reuse (Wilkinson et al., 2016), and iterative review. At the process level, this formative case suggests that operationalizing fairness in digital health depends not only on existing standards but also on viable institutional structures for acting on them.

### 6.5 Limitations and future research

This study has five main limitations. First, it is based on a single-case study in an early deployment and redesign context. This provided a useful setting for examining fairness knowledge translation before the routines and responsibilities were stabilized, but it also limits the transferability of these findings to more mature systems or differently structured organizational settings. Team roles changed during the project, and some participants had joined only recently; responses may therefore reflect fluid role boundaries and asymmetric organizational memory, which can also arise in real-world implementation settings. Because recruitment was not extended in response to emerging sub-codes, code saturation was not established and the inductive sub-codes should not be considered exhaustive. Second, although F2A was developed independently of HealthEmove, the lead author's role in framework development, formative evaluation, primary coding, and consortium meetings introduces the potential for researcher-led self-evaluation, confirmation bias, and social-desirability bias. In this small, embedded team, courtesy effects may also have contributed to favorable ratings. The qualitative findings should therefore be interpreted as a framework-guided account of this embedded case. Three CRU respondents were co-authors; excluding them produced slightly lower but still favorable mean scores. Because the remaining respondents were also project stakeholders, the ratings may nonetheless be optimistic. CRU scores should therefore be interpreted as formative, self-reported indicators of perceived interpretability and fit, not evidence of framework validation or subsequent use. Third, the Hypothetical Fairness Risk Scenario prioritized a common, accessible elicitation context over modality-level correspondence with the anticipated HealthEmove feature. Its deliberately simplified design, including reliance on a single accuracy-based cue, may have shaped participants' interpretation of the stated risk and limits the ecological validity of the scenario-based findings. Fourth, the study focuses on how fairness-related knowledge is accessed, interpreted, and operationalized, not on downstream behavioral or outcome-level effects. The findings therefore support claims about practical translation processes within this case, not about improved fairness outcomes in deployment. Fifth, the study included professional stakeholders only; intended HealthEmove users and community representatives did not participate. The resulting guidance was therefore not evaluated against community priorities and may omit harms evident through lived experience.

These limitations point to several directions for future research: (i) longitudinal work could examine how fairness knowledge translation changes as projects move from early design to implementation, monitoring, sustainment, and maintenance; (ii) comparative studies across projects, domains, and organizational settings could assess whether the same prompt structures and responsibility-mapping procedures remain actionable under different governance arrangements, technical maturities, and institutional conditions, and how local facilitation, documented adaptation, and alignment with reporting requirements affect portability; (iii) future work could evaluate F2A across multiple settings and examine multiple role-specific instantiations rather than a single focal role. A multi-role evaluation would minimally require artifacts for roles with different responsibilities, assessment of each artifact by the target role holder and relevant collaborating roles, and observation of actual use over time rather than reliance on perceived CRU ratings alone. A concrete next step is to recruit intended users and community representatives to review responsibility mappings and co-design the resulting artifacts, allowing the guidance to be assessed against community priorities and harms identified through lived experience; (iv) multi-country studies could clarify how regulatory environments and health system characteristics shape the operationalization of fairness; (v) studies of deployed human–AI workflows could translate responsibilities mapped through F2A into operational runbooks and escalation triggers, develop team-readiness metrics, and use interaction traces to evaluate reliance calibration, error recovery, and learning over time; and (vi) later-stage studies could test independent application by external intended users after standardized onboarding, using artifact observation, task-based measures such as completion time and error rates, and pre/post-assessments of role clarity and documentation quality to assess actual use, examine whether responsibility mapping reduces ethical offloading and preserves continuity as roles and decision authority change, and assess whether improvements in role clarity, documentation, and coordination are associated with sustained fairness practices and, eventually, improved downstream outcomes.

### 6.6 Broader contribution

The broader contribution of this formative case lies in examining fairness not only as a property to specify but also as work requiring project-level organization. F2A structured analysis of how this work was distributed and interpreted and how its prompts could be converted into role-specific guidance, without defining substantive fairness requirements.

This broader framing also identifies the study's potential relevance beyond digital health. The specific prompts, artifacts, and constraints examined here are domain-bound, but role differentiation, distributed responsibility, and cross-stakeholder coordination may also arise in other domains. The HealthEmove case therefore provides a process-level proposition that fairness work may become more actionable when knowledge translation is treated as an organizational problem rather than a purely technical one. Examining this proposition in other settings would require context-specific instantiation, renewed responsibility mapping, local facilitation, and alignment with applicable governance and reporting requirements. A concise implementation and maintenance note is provided in Supplementary material E.

## 7 Conclusion

Our study formatively evaluated the F2A framework in an early-stage live digital health project and conducted a proof-of-concept instantiation for the Junior Researcher role. In HealthEmove, fairness-related knowledge was present but asymmetrically distributed, only partly embedded in shared routines, and interpreted differently across roles depending on decision authority, proximity to implementation, and access to relevant knowledge. The favorable CRU ratings are interpreted as potentially optimistic indicators of perceived fit, while responsibility mapping made cross-role dependencies explicit and supported the development of a role-specific guidance artifact. Collectively, these findings support three contributions: empirical application of F2A in an early-stage live project setting, formative evaluation through prompt-level assessment, and a proof-of-concept application of a responsibility-mapping instantiation procedure to one role. Rather than defining the correct fairness solution or evaluating downstream fairness outcomes, our study frames algorithmic fairness as a knowledge translation problem and illustrates, within this formative case, a potential process-level approach for examining how fairness-related work is organized across roles in multi-stakeholder digital health projects.

## Data Availability

The datasets presented in this article are not readily available because the primary qualitative and quantitative interview responses are not publicly available due to risks of indirect participant identification, while the licensed third-party microdata cannot be redistributed by the authors. Aggregated quantitative summaries, the interview instrument, framework materials, RACI task matrix, role-specific guidance artifact, and redacted analysis code are included in the article and Supplementary materials. Access to additional de-identified primary materials is subject to applicable institutional requirements, participant consent, and project confidentiality constraints. The secondary microdata used to construct the hypothetical fairness risk scenario were obtained from the UNHCR Microdata Library (https://microdata.unhcr.org). Other researchers may apply for access through the UNHCR Microdata Library catalog under reference UNHCR_EU_2023_MSNA. Further code details are subject to the UNHCR Microdata Library Terms of Use and applicable access restrictions. Requests to access the datasets should be directed to Primary interview data: Sara Altamirano, s.e.altamirano@uva.nl. Secondary UNHCR MSNA 2023 microdata: https://microdata.unhcr.org.

## Footnotes

1
https://www.healthemove.org/

2
https://www.digitalevoordeur.amsterdam/

3 Within HealthEmove, *users* refers to intended app users and *patients* to those same individuals in care relationships with healthcare professionals. HealthEmove does not provide clinical care.

4
https://openresearch.amsterdam/en/page/99463/digitale-voordeur—platform-zorg-en-welzijn

5
https://microdata.unhcr.org/index.php/terms-of-use
