# Supplementary material for: Translating algorithmic fairness into health practice: formative evaluation of the fairness-to-action framework
Source: Front Artif Intell. 2026 Sep 21;9:1911214. doi: 10.3389/frai.2026.1911214 (PMC13635205; doi:10.3389/frai.2026.1911214)
Supplement: Supplementary file 1 [file Data_Sheet_1.pdf]

# *Supplementary Material A: Analysis and Coding Protocol for Interview Data*

## 1 MAIN ANALYTIC STRATEGY

### 1.1 Overall analytic approach

The study used an exploratory mixed-methods design, combining qualitative analysis with descriptive quantitative elements, to support formative evaluation of the Fairness-to-Action (F2A) framework. Interview data and Clarity, Relevance, and Usability (CRU) scores were analyzed separately using procedures appropriate to each data type. Quantitative analysis described participant evaluations of F2A item clarity, relevance, and usability. Qualitative analysis examined how participants interpreted, appraised, and contextualized the F2A items in relation to their roles and project responsibilities.

The qualitative and quantitative components were reported separately in the Results section and considered together only at the level of interpretation in the Discussion. No formal mixed-method integration procedures, such as joint displays, merged datasets, or statistical integration of qualitative and quantitative findings, were applied. This approach supported transparent reporting and aligned with the formative evaluation focus of the study.

### 1.2 Data preparation

**Interview and written-response data.** Interviews were conducted orally or completed in written form, depending on participant preference and availability. Oral interviews were transcribed using Amberscript or Microsoft Teams transcription, with manual correction where needed. Transcripts were cleaned for readability only. Meaning was not intentionally altered during cleaning.

Tangents, hesitations, backtracking, repetitions, and clarifications were retained where analytically relevant or marked where needed. Clarifications provided during interviews were retained in the transcript when relevant for interpreting participant responses. All transcripts and written responses were pseudonymized before analysis and reported without participant names, with measures to reduce attribution risk in the small project setting.

**Quantitative data.** Questionnaire responses were stored in a long-format spreadsheet. Each row represented one participant response to one questionnaire item. Variables included participant identifier, role group, question number, question label, question code, domain, score type, score, raw answer, answer length, and notes.

CRU scores were stored for all scored F2A items, corresponding to items 1 to 22. Single-score items from the Framework Assessment, Scenario-Based Evaluation, and Questionnaire Design sections were also stored where applicable. Written answers were stored verbatim. No qualitative coding or interpretation was conducted in the spreadsheet.

### 1.3 Coding structure in ATLAS.ti

Qualitative analysis was conducted in ATLAS.ti. One ATLAS.ti document was created per participant. Each document contained the full interview transcript or written interview text. Only responses to F2A items 1 to 22 were included in the primary item-level coding process.

A fixed set of 22 parent codes aligned with the F2A framework was used. The definitions of the 22 parent codes remained fixed throughout analysis. Subcodes were created by the primary researcher after cross-case review when recurrent patterns or analytically important distinctions required clarification.

**Parent codes.**

```
WHAT domain
01_WHAT_Knowledge_Availability
02_WHAT_Responsibility_Holders
03_WHAT_Knowledge_Sources
04_WHAT_Knowledge_Access
05_WHAT_Knowledge_Effects

HOW domain
06_HOW_Inquiry_Process
07_HOW_Knowledge_Synthesis
08_HOW_Fairness_Tools
09_HOW_Needs_Identification
10_HOW_Knowledge_Selection
11_HOW_Knowledge_Adaptation
12_HOW_Barrier_Assessment
13_HOW_Intervention_Implementation
14_HOW_Use_Monitoring
15_HOW_Outcome_Evaluation
16_HOW_Use_Sustainment

WHY domain
17_WHY_Fairness_Framing
18_WHY_Action_Gap
19_WHY_Routine_Reporting
20_WHY_Reputational_Concerns
21_WHY_Metric_Dominance
22_WHY_Disciplinary_Divides
```

### 1.4 Segmentation and coding procedure

Coding was conducted in two passes.

**First pass: segmentation and parent coding.** Segmentation and primary coding were performed by the primary researcher. Responses to F2A items 1 to 22 were processed in questionnaire order. For each F2A item, one or more quotations were created as needed.

A quotation was defined as a segment of text expressing one coherent idea relevant to a single F2A item. Segments could span one sentence or multiple sentences. A new segment was created when the participant introduced a new analytic idea relevant to the question. When uncertain, responses were split into separate quotations rather than combined into one. During this pass, each quotation was assigned the parent code corresponding to the F2A item. No subcodes were created.

**Second pass: cross-case review and pattern refinement.** After all documents were segmented and parent coded, quotations were compared across participants within each parent code. Subcodes were created and applied when recurrent patterns or analytically important distinctions required clarification. Quotations were not split, merged, resized, or deleted during this pass. The 22 parent codes and their definitions remained in the code system.

Code saturation was not used as an analytic stopping criterion. The case-bounded, role-stratified sample and 22-prompt coding frame were specified before analysis, and recruitment was not extended in response to emerging subcodes. The analysis sought framework-guided coverage and cross-role variation rather than exhaustive saturation.

## 1.5 Complementary questionnaire sections

Framework Assessment, Scenario-Based Evaluation, and Questionnaire Design sections were analyzed as complementary components of the overall evaluation. They were not treated as separate supplementary datasets. These sections were considered substantively important alongside the 22 F2A item assessments, while addressing distinct evaluation purposes.

Framework Assessment items examined participant views on the overall structure, fit, completeness, usefulness, and feasibility of the F2A framework. Scenario-Based Evaluation items examined perceived seriousness, confidence, need for action, coordination demands, equity implications, and trust implications in the presented fairness risk scenario. Questionnaire Design items examined participant views on clarity, ease of use, workload, realism, intuitive use, relevance, and technical functioning of the questionnaire.

Quantitative responses from these sections were analyzed descriptively in the spreadsheet using summary statistics and score distributions. Open-text comments from these sections were reviewed qualitatively outside the primary ATLAS.ti item-level coding framework to identify recurring explanations, concerns, and suggestions. These sections were reported in the Results under Framework Evaluation and used to complement the item-level findings from the 22 F2A questions. They were analyzed separately from the main F2A coding framework unless comments explicitly referred to one or more F2A items.

## 1.6 Quantitative analysis of CRU scores

Quantitative analysis focused on descriptive summaries of participant scores for clarity, relevance, and usability. Analyses were conducted for F2A items 1 to 22. Results were summarized using appropriate descriptive statistics, including means, medians where informative, minimum and maximum values, score dispersion, and frequency distributions.

Scores were examined by individual item and by F2A domain. Cross-role CRU comparisons were not performed because individual roles were generally represented by one participant and the broader role groups were too small for reliable comparison. Any CRU score of 1 was prespecified to trigger review of the corresponding item and possible prompt revision. Quantitative analysis was used to describe item-level rating distributions and determine whether the pre-specified score-1 review rule was triggered. Observed differences between items were not interpreted as reliable. Framework Assessment, Scenario-Based

Evaluation, and Questionnaire Design scores were also analyzed descriptively in the spreadsheet. No inferential statistical testing was planned because of the formative purpose, limited sample size, and non-comparative design.

### 1.7 Qualitative analysis

ATLAS.ti was used to analyze quotations for F2A items 1 to 22. All coded material was eligible for qualitative analysis and was not selected on the basis of score thresholds. Quotations were reviewed to identify recurring mechanisms, constraints, interpretations, tensions, and practical considerations reported by participants.

Code-document tables and targeted code comparisons were used to examine how patterns were distributed across participants and role groups. Analysis focused on both recurring patterns and meaningful divergence. Particular attention was given to how participants understood each item, perceived usefulness or limitations, implementation barriers, examples of existing practices, aspirational practices, and role-specific needs or concerns.

### 1.8 Memoing

Memos were written after completion of parent coding and during or after cross-case review.

A code memo was written for each of the 22 parent codes. These memos summarized recurring patterns, distinctions, tensions, notable absences, and role patterns within each parent code.

Three domain memos synthesized patterns across the WHAT, HOW, and WHY domains. These memos integrated relevant item-level memos, surfaced cross-item patterns and gaps, and helped prepare the logic of the Results section.

Document memos summarized each participant's role context and notable, distinctive, or deviant responses.

The project memo recorded analytic decisions, coding rules, clarifications of parent-code and subcode boundaries, and contextual notes related to the primary researcher's framework-development work and participation in consortium meetings. Code memos and domain memos served as the primary qualitative synthesis tools for manuscript development.

### 1.9 Cross-role comparison

After primary coding, responses were compared across participant role groups. Comparisons focused on similarities and differences in how roles understood, valued, questioned, or prioritized F2A items. Attention was given to recurring coordination issues, responsibility ambiguity, dependencies, and role-specific needs where evident. These comparisons were descriptive and exploratory. They were not used to rank roles or evaluate role performance.

### 1.10 Output and reporting

Results were reported primarily by F2A domain and by item where relevant. Item numbers and short labels were used when useful for clarity, for example, `06_HOW_Inquiry_Process`. Quantitative findings summarized CRU score patterns. Qualitative findings described how participants interpreted and evaluated the items in practice. Short excerpts were included where they added analytic value and where attribution risk was low; excerpts were reported without participant names.

Findings explicitly distinguished between established practices, aspirational practices, uncertainties, and identified gaps. Role-specific implications for improving the framework, checklists, and responsibility mappings were reported where relevant.

### 1.11 Interpretive stance

Quantitative and qualitative findings were not statistically or procedurally merged. Instead, both were considered together only at the level of overall interpretation in the Discussion section. This approach supported transparent reporting and aligned with the formative evaluation focus of the study.

## 2 SECONDARY CODER INSTRUCTIONS

### 2.1 Purpose

The secondary coding procedure was designed to support analytic credibility through secondary review of the primary coding decisions. The secondary coder assessed whether the existing segments and applied parent codes or subcodes were appropriate, identified unclear code boundaries, and flagged ambiguous segments for discussion.

### 2.2 Materials

The secondary coder received an ATLAS.ti project bundle prepared by the primary researcher, the interview questionnaire, the code list, and a shared disagreement log. The secondary coder was a co-author but was not involved in developing F2A, HealthEmove, data collection, or primary coding. The ATLAS.ti project contained transcripts that had already been segmented and coded by the primary researcher.

### 2.3 Task

The secondary coder reviewed the existing coded material without modifying the ATLAS.ti project file. The task was to agree or disagree with the segments selected and the codes applied. If the coder agreed, no action was needed. If the coder disagreed, the disagreement was recorded in the shared log for subsequent discussion with the primary researcher.

### 2.4 Step-by-step procedure

1. Open the ATLAS.ti project and use the existing documents for review only.
2. Read the interview questionnaire in full to understand the structure and context of the data.
3. Read the code list and code definitions before reviewing transcripts and coding.
4. Review each response and its existing quotations one by one.
5. Check whether relevant material was appropriately included and segmented and whether the applied parent code or subcode fits its meaning.
6. If the coding fits, take no action.
7. If the coding does not fit, record the disagreement in the shared disagreement log.

### 2.5 Recording disagreements

For each observation, the secondary coder recorded the participant identifier, question number, relevant segment or note, and rationale. Observations could concern code fit, segment inclusion or clarity,

segmentation, or whether a new subcode might be warranted. During secondary review, proposals for new subcodes were considered only when the pattern appeared across three or more transcripts.

## 2.6 Rules

The secondary coder did not create, delete, merge, split, or resize segments; add, remove, or rename parent codes; create subcodes independently; or return a modified ATLAS.ti project file. Disagreements were resolved through discussion with the primary researcher. Final coding decisions and analysis remained the responsibility of the primary researcher.

## 2.7 After review

The secondary coder and primary researcher discussed disagreements only. These discussions were used to clarify code definitions, refine code boundaries, and decide whether any recoding, re-segmentation, or new subcode creation was needed. The secondary coder could also comment on primary researcher memos where clarification or disagreement was relevant.

## 2.8 Review outcomes

The secondary coder reviewed all 241 open-ended responses and recorded 84 question-level observations concerning 83 responses. Twenty-seven observations were classified as coding disagreements requiring resolution: 10 concerned code assignment or interpretation, 10 the appropriate coding level, and seven segment inclusion or analytic scope. The remaining 57 concerned clarifications, supplementary coding suggestions, or confirmations of the existing coding.

All 27 disagreements were discussed and resolved. In the final coding, 12 resolutions aligned with the secondary coder's position, seven reflected partial or alternative resolutions, and the primary researcher's position was retained in eight cases. These outcomes describe the review and reconciliation process and are not inter-coder agreement statistics. Neither percentage agreement nor an inter-coder reliability coefficient was calculated because the review did not produce paired independent coding decisions at a common segment-code unit.

Table S1: De-identified resolutions of the 27 coding disagreements

| ID | Nature | Resolution | Resulting change |
| --- | --- | --- | --- |
| D01 | Code assignment or interpretation | Secondary position adopted | Reassigned the disputed passage from practical criteria to benchmark comparison. |
| D02 | Code assignment or interpretation | Alternative resolution | Separated authority and knowledge barriers; coded the authority issue as tradeoffs or tensions and retained the knowledge gap. |
| D03 | Code assignment or interpretation | Alternative resolution | Coded the audit and dashboard passage as monitoring checkpoints and retained the remaining material at the parent-code level. |
| D04 | Segment inclusion or analytic scope | Secondary position adopted | Retained the reporting-trigger and learning-focus segments and excluded the remaining material from this item. |
| D05 | Coding level | Secondary position adopted | Moved the quotation from a subcode to the corresponding parent code. |
| D06 | Code assignment or interpretation | Secondary position adopted | Coded the complete passage as indicating existing knowledge rather than a need for knowledge. |
| D07 | Segment inclusion or analytic scope | Primary position retained | Retained all disputed passages; moved the first passage from informal inquiry to the parent inquiry-process code and retained the proposed expert advice as informal inquiry. |

Continued on next page

Table S1 continued

| ID | Nature | Resolution | Resulting change |
|---|---|---|---|
| D08 | Segment inclusion or analytic scope | Primary position retained | Retained the three disputed passages and their existing coding. |
| D09 | Segment inclusion or analytic scope | Primary position retained | Retained both disputed passages; moved the first from awareness actions to the parent intervention-implementation code and retained the second at the parent level. |
| D10 | Code assignment or interpretation | Secondary position adopted | Applied the ethical-priority subcode to one passage, the parent framing code to the mixed framing, and excluded the additional disputed passage. |
| D11 | Segment inclusion or analytic scope | Alternative resolution | Added parent-level metric-dominance coding while retaining the overlapping efficiency-pressure coding and disputed material. |
| D12 | Coding level | Secondary position adopted | Moved the absence statement to the parent inquiry-process code while retaining the regulatory lifecycle passage as structured inquiry. |
| D13 | Code assignment or interpretation | Primary position retained | Retained the substantive interpretation; the code labels were updated from benchmarks needed and testing tools to general frameworks and technical tools. |
| D14 | Coding level | Secondary position adopted | Moved the data-availability barrier from the knowledge-gaps subcode to the parent barrier-assessment code. |
| D15 | Code assignment or interpretation | Alternative resolution | Added nested existing-knowledge coding while retaining the broader passage coded as a need for knowledge. |
| D16 | Coding level | Secondary position adopted | Moved the data-gap passage to the parent barrier-assessment code. |
| D17 | Coding level | Secondary position adopted | Added a parent-level outcome-evaluation quotation while retaining the examples-needed subcode. |
| D18 | Segment inclusion or analytic scope | Alternative resolution | Added the substantive design-changes segment while retaining the coded clarification question. |
| D19 | Code assignment or interpretation | Alternative resolution | Moved the passage from the testing-signals subcode to the parent needs-identification code; the proposed cross-item codes were not applied. |
| D20 | Code assignment or interpretation | Alternative resolution | Added time-constraints coding while retaining the tradeoffs-or-tensions interpretation. |
| D21 | Coding level | Secondary position adopted | Moved the quotation to the parent outcome-evaluation code; the proposed cross-item code was not applied. |
| D22 | Segment inclusion or analytic scope | Primary position retained | Retained all three passages; changed one passage from user complaints to monitoring checkpoints, while the other two remained coded as monitoring checkpoints. |
| D23 | Code assignment or interpretation | Primary position retained | Retained structured-inquiry coding for the proposed knowledge base. |
| D24 | Coding level | Primary position retained | Retained the tradeoffs-or-tensions subcode rather than moving the passage to the parent barrier-assessment code. |
| D25 | Coding level | Secondary position adopted | Moved the passage to the parent use-monitoring code. |
| D26 | Coding level | Primary position retained | Retained the examples-needed subcode rather than moving the passage to the parent outcome-evaluation code. |
| D27 | Coding level | Secondary position adopted | Moved the passage to the parent metric-dominance code. |

## 2.9 Final Codebook

### 2.9.1 WHAT domain

**01_WHAT_Knowledge_Availability**

General statements about whether fairness knowledge exists, is recognized, or is perceived to exist, including assumed availability.

**01_WHAT_Knowledge_Availability_have**
Fairness knowledge is currently available or present.

**01_WHAT_Knowledge_Availability_need**
Fairness knowledge is missing or needed.

**02_WHAT_Responsibility_Holders**
Who holds or should hold fairness knowledge or expertise.

**03_WHAT_Knowledge_Sources**
Where fairness knowledge comes from in general.

**03_WHAT_Knowledge_Sources_internal**
Knowledge from internal team members or roles.

**03_WHAT_Knowledge_Sources_external**
Knowledge from external experts or organizations.

**03_WHAT_Knowledge_Sources_regulatory**
Knowledge from laws, regulation, or compliance guidance.

**04_WHAT_Knowledge_Access**
How people access fairness knowledge or support.

**04_WHAT_Knowledge_Access_current**
How access to fairness knowledge currently works.

**04_WHAT_Knowledge_Access_desired**
How access to fairness knowledge should work.

**05_WHAT_Knowledge_Effects**
Whether fairness knowledge affects project decisions.

**05_WHAT_Knowledge_Effects_design_changes**
Knowledge leads to design or management changes.

**05_WHAT_Knowledge_Effects_model_selection**
Knowledge influences model or tool choice.

**05_WHAT_Knowledge_Effects_no_change**
Fairness knowledge is acknowledged but does not lead to changes in design, management, or model choice.

### 2.9.2 HOW domain

**06_HOW_Inquiry_Process**
How fairness questions are raised, either in practice or as an explicitly described ideal or expectation, including desired processes.

**06_HOW_Inquiry_Process_informal**
Fairness questions are raised ad hoc or individually, with no process in place.

**06_HOW_Inquiry_Process_structured**
Fairness inquiry is described through formal, regulatory, or lifecycle mechanisms, even if only as a desired or required process and not yet implemented.

**07_HOW_Knowledge_Synthesis**
How fairness knowledge is combined across sources.

**07_HOW_Knowledge_Synthesis_central_coordination**
One role coordinates or synthesizes knowledge.

**07_HOW_Knowledge_Synthesis_team_discussion**
Knowledge is synthesized through team discussion.

**07_HOW_Knowledge_Synthesis_not_yet**
Synthesis of fairness knowledge is not currently happening or not yet established.

**07_HOW_Knowledge_Synthesis_authority_dependence**
Synthesis requires mandate, approval, or pressure from senior or authoritative roles to occur.

**08_HOW_Fairness_Tools**
Tools or resources to apply fairness in practice.

**08_HOW_Fairness_Tools_general_frameworks**
Need or gap for benchmarks, standards, examples, or reference cases to guide fairness work.

**08_HOW_Fairness_Tools_technical_tools**
Technical tools or tests for fairness assessment, such as checklists, fairness metrics, or templates.

**09_HOW_Needs_Identification**
How fairness issues are detected.

**09_HOW_Needs_Identification_user_feedback**
Issues identified through users of HealthEmove or affected groups.

**09_HOW_Needs_Identification_testing_signals**
Fairness issues identified through testing or evaluation results.

**09_HOW_Needs_Identification_unpredictable**
Fairness issues are described as difficult or impossible to identify in advance, even if partial signals exist.

**10_HOW_Knowledge_Selection**
How fairness methods or approaches are chosen.

**10_HOW_Knowledge_Selection_practical_criteria**
Selection based on feasibility or usability.

**10_HOW_Knowledge_Selection_benchmark_comparison**
Selection based on formal references such as standards, guidelines, regulatory frameworks, or structured artifacts used to justify or compare choices.

**11_HOW_Knowledge_Adaptation**
Adapting fairness ideas to context.

**11_HOW_Knowledge_Adaptation_user_context**
Adaptation to users, populations, or settings.

**11_HOW_Knowledge_Adaptation_tradeoffs**
Accepting or managing fairness trade-offs between competing goals.

**11_HOW_Knowledge_Adaptation_practice_based**
Adaptation of fairness ideas through everyday project practices, informal feedback, or learning by doing.

**11_HOW_Knowledge_Adaptation_formal_methods**
Adaptation of fairness ideas through formal guidelines, tools, checklists, standards, or predefined fairness frameworks.

**12_HOW_Barrier_Assessment**
Barriers to acting on fairness goals.

**12_HOW_Barrier_Assessment_knowledge_gaps**
Barriers due to lack of understanding or skills.

**12_HOW_Barrier_Assessment_tradeoffs_tensions**
Conflicting goals, priorities, interests, or unclear responsibility or authority.

**12_HOW_Barrier_Assessment_time_constraints**
Limited time or capacity.

**13_HOW_Intervention_Implementation**
Concrete actions to improve fairness.

**13_HOW_Intervention_Implementation_awareness_actions**
Actions to raise awareness or understanding.

**13_HOW_Intervention_Implementation_testing_actions**
Testing or evaluation actions.

**13_HOW_Intervention_Implementation_data_requirements**
Data needs to implement fairness actions.

**14_HOW_Use_Monitoring**
Tracking fairness over time, beyond one-off checks.

**14_HOW_Use_Monitoring_checkpoints**
Periodic reviews or checkpoints.

**14_HOW_Use_Monitoring_user_feedback**
Monitoring through complaints or reported issues.

**15_HOW_Outcome_Evaluation**
Learning from past fairness outcomes, based on prior experience.

**15_HOW_Outcome_Evaluation_examples_needed**
Need for concrete examples, cases, or prior experiences to learn from.

**16_HOW_Use_Sustainment**
Maintaining fairness practices over time.

**16_HOW_Use_Sustainment_documentation_handover**
Continuity through documentation or role transfer.

**16_HOW_Use_Sustainment_values_embedding**
Embedding fairness as a core value, principle, or priority across the project or organization.

### 2.9.3 WHY domain

**17_WHY_Fairness_Framing**
How fairness is conceptually framed.

**17_WHY_Fairness_Framing_ethical_priority**
Fairness as an ethical or social responsibility.

**17_WHY_Fairness_Framing_technical_support**
Fairness framed primarily as a technical issue, with ethical or social aspects treated as secondary or supportive.

**18_WHY_Action_Gap**
Gap between discussion and action.

**18_WHY_Action_Gap_discussion_mostly**
Fairness remains mostly conceptual.

**18_WHY_Action_Gap_resource_limits**
Action limited by resources, including knowledge, time, or capacity.

**19_WHY_Routine_Reporting**
Purpose of reporting fairness work.

**19_WHY_Routine_Reporting_learning_focus**
Reporting for learning and improvement.

**19_WHY_Routine_Reporting_reporting_trigger**
Reporting driven by external requirements.

**20_WHY_Reputational_Concerns**
Fear of reputational consequences.

**20_WHY_Reputational_Concerns_non_disclosure**
Withholding negative results or sensitive findings.

**20_WHY_Reputational_Concerns_time_costs**
Costs of disclosure or remediation.

**21_WHY_Metric_Dominance**
Weighing performance metrics against fairness considerations, including implicit prioritization.

**21_WHY_Metric_Dominance_efficiency_pressure**
Pressure for speed or efficiency.

**21_WHY_Metric_Dominance_external_accountability**
Fairness prioritized when externally assessed.

**22_WHY_Disciplinary_Divides**
Collaboration challenges across roles or organizations.

**22_WHY_Disciplinary_Divides_misaligned_priorities**
Different goals or understandings across partners.

**22_WHY_Disciplinary_Divides_early_alignment**
Early coordination to reduce friction.

### Rules of thumb for first pass

- Use one parent code per F2A item.
- Segment by analytic idea, not by sentence.
- Code only material relevant to the F2A item.
- Use the fewest quotations needed to capture distinct ideas.
- Do not create subcodes in the first pass.
- Keep "see above" as its own quotation and use a quotation comment for the reference.
- Do not edit, rewrite, or clean text inside ATLAS.ti.
- Do not code Framework Assessment, Scenario-Based Evaluation, or Questionnaire Design sections during the primary item-level coding process.
- When a response introduced more than one distinct analytic idea, split it into separate quotations.

**Rules of thumb for second pass**

- During the second pass, apply subcodes only to quotations that had first been assigned a parent code.
- Create subcodes when a recurrent pattern or analytically important distinction adds analytic value.
- Use subcodes to distinguish analytic differences, not to restate the question.
- Apply the most specific applicable code within the parent-code hierarchy; retain the parent code alone when no subcode fits.
- Retain all 22 parent codes and their definitions in the code system; do not remove or rename them.
- Do not split or merge quotations during the second pass.
- Use code lists and quotation retrieval to review subcoded quotations; use co-occurrence tables where multiple codes were assigned.
- Write memos to document the analytic pattern captured by each subcode where useful.
- If a subcode does not add analytic value, do not use it.

## 3 GLOSSARY

**Parent code**
A code corresponding directly to a single F2A questionnaire item, applied during first-pass coding to anchor quotations to that item.

**Subcode**
An analytic code applied during the second pass to refine or distinguish patterns within quotations initially coded with the corresponding parent code.

**Segment**
A continuous span of text expressing one coherent idea and selected as a unit for coding. Segments were kept fixed during second-pass subcoding but could be revised during secondary-coder reconciliation. In ATLAS.ti, segments are called quotations.

**Document**
A single interview transcript or written questionnaire response associated with one participant and imported into ATLAS.ti.

**Quotation**
A selected segment of text in ATLAS.ti to which one or more codes are applied.

**Co-coding**
The practice of applying multiple codes to the same quotation to enable analytic refinement without changing the parent-code structure.

**Code co-occurrence table**
An ATLAS.ti analysis table showing quotations where two or more codes are applied together, used to examine co-coded patterns.

## 4 TRANSCRIPT CLEANING GUIDELINES

**Scope and purpose.** Transcripts were cleaned for qualitative coding, not for stylistic polish or discourse analysis. The aim was readability and analytic usability while preserving meaning. Coding was semantic and question driven.

**Participant speech.** Participant wording was kept verbatim. No paraphrasing, synonym replacement, or rephrasing was performed. Word order and meaning were preserved. Hesitations, uncertainty markers, and repetitions could remain. Grammar and fluency were not corrected. Consecutive participant turns could be concatenated if they expressed a single stable idea. Bracketed clarifications could be added only to support comprehension and were clearly marked. Bracketed clarifications were excluded from verbatim quotations.

**Interviewer speech.** Fillers and acknowledgements were removed. Procedural or instructional talk was removed. Clarifications were removed if they did not add substantive meaning. Each question was retained once, without repetition.

**Consolidation rules.** Consolidation was done only by deletion and concatenation, not by rewriting. Consolidation was allowed when the participant maintained a single position throughout the response. Consolidation was not allowed when meaning shifted because of interviewer prompts, clarification, or challenge. Interactional structure was preserved when necessary for understanding participant reasoning.

**Brackets and annotations.** Brackets were used only to clarify references, roles, or tools. Bracketed text did not replace participant wording and was excluded from verbatim quotations.

**Quoting.** Verbatim quotations were taken from the original transcript or written response. Cleaned transcripts were used for coding and analysis only.

**Stopping rule.** Cleaning stopped once the text was readable and codeable. Messiness was acceptable if meaning was preserved. No further edits were made once a segment was coded.
