# Supplementary material for: Translating algorithmic fairness into health practice: formative evaluation of the fairness-to-action framework
Source: Front Artif Intell. 2026 Sep 21;9:1911214. doi: 10.3389/frai.2026.1911214 (PMC13635205; doi:10.3389/frai.2026.1911214)
Supplement: Supplementary file 2 [file Data_Sheet_2.pdf]

frontiers

Supplementary Material B: Data Protection, Ethics Documentation, and Data Management Plan

1 PURPOSE OF THIS SUPPLEMENTARY FILE

This supplementary file summarizes the data protection, ethics documentation, and data management procedures used in the study. It is provided for transparency and reproducibility. It does not constitute a legal assessment of GDPR compliance.

2 DATA PROTECTION AND ETHICS DOCUMENTATION CHECKLIST

Table S1: Data protection and ethics documentation checklist.

Item	Status	Documentation / notes
Research purpose explained to participants	Completed	Information sheet described the study purpose and the use of interview responses for research.
Participant information sheet provided	Completed	Information sheet included purpose, invitation rationale, participation format, confidentiality, data protection, consent, and researcher contact details.
Informed consent obtained	Completed	Participants provided consent before participation by submitting written responses or signing/initialing information sheets, depending on completion mode.
Voluntary participation stated	Completed	Participants were informed that participation was voluntary and that they could skip questions or stop at any time without consequence.
Participant population assessed	Completed	Participants were professional stakeholders only; no patients or vulnerable populations were included as research participants.
Institutional ethics consultation	Completed	The Ethics Committee for Information Sciences (ECIS), Faculty of Science, University of Amsterdam, advised through a series of consultations that formal ethical review was not required for this non-invasive study involving adult professional stakeholders providing expert opinions.

Continued on next page

Item	Status	Documentation / notes
Data categories assessed	Completed	Data consisted of professional stakeholder responses, CRU scores, additional questionnaire ratings, role-related project information, analytic memos, coding outputs, and pseudonymized participant IDs. No patient-level data, identifiable health records, medical data from participants, direct identifiers, or special-category personal data were retained in the analysis files.
Data minimization applied	Completed	Only data needed for the research questions were collected and analyzed.
Pseudonymization prior to analysis	Completed	Responses were organized using pseudonymized participant identifiers before analysis.
Attribution-risk reduction in reporting	Completed	Findings are reported without participant names, using broad role groupings and excerpts selected to reduce attribution risk.
Restricted data access	Completed	Study data were stored in a restricted-access University of Amsterdam research environment.
Interview recordings handled separately	Completed	Recordings, where applicable, were stored separately from transcripts, written responses, and extracted scores.
Transcription and video-conferencing services assessed	Completed	Microsoft Teams and Amberscript were used where applicable only for interview facilitation, recording, or transcription. Access was restricted to the primary researcher through access-controlled accounts. Service-generated files were deleted where applicable after transcription or verification, and analysis files were pseudonymized before analysis.
Use of external data source documented	Completed	UNHCR Microdata Library data used for the hypothetical fairness risk scenario were accessed and analyzed according to the applicable Terms of Use and only for approved scientific research purposes.
Raw data sharing assessed	Completed	Raw interview and questionnaire data are not made public because the small project setting creates a risk of indirect identification. Aggregated summaries and study materials are provided where possible.
Potential researcher bias documented	Completed	The primary researcher's contextual involvement was disclosed; reflexive memos and secondary-coder review were used to support analytic credibility.
Co-author participant roles disclosed	Completed	Co-author participants contributed study responses as domain experts but were not involved in coding, data analysis, or interpretation of empirical findings.

3 DATA MANAGEMENT PLAN

3.1 Data collected

The study generated written questionnaire responses, interview recordings where applicable, transcripts, extracted Clarity, Relevance, and Usability (CRU) scores, additional questionnaire ratings, analytic memos, coding outputs, and pseudonymized participant IDs.

3.2 Data not collected for analysis

The study did not collect patient-level data, identifiable health records, medical data from participants, sensitive or special-category personal data, or direct identifiers for analysis files.

3.3 Participant information and consent

Participants received an information sheet before participation. Participation was voluntary, and participants could skip questions or stop participation at any time without explanation or consequence. Consent was documented through signed or initialed information sheets before participation; for written responses, submission also confirmed consent in accordance with the participant information sheet. Participants consented to the use of anonymized quotations in research outputs.

3.4 Storage and access

Raw materials were stored in a restricted-access University of Amsterdam research environment. Access was limited to the research team. Signed or initialed consent materials were stored separately from response data. Interview recordings, where applicable, were stored separately from transcripts, written responses, and extracted scores.

3.5 Transcription and video-conferencing services

Where interviews were conducted or transcribed using Microsoft Teams or Amberscript, these services were used only for interview facilitation, recording, or transcription. Access to recordings and transcripts was restricted to the primary researcher through access-controlled accounts. Recordings and transcripts generated through these services were downloaded or reviewed only for research purposes and were not shared outside the research process. After transcription and quality checking, recordings were stored in the restricted-access University of Amsterdam research environment separately from transcripts, written responses, and extracted scores. Service-generated files were deleted where applicable after they were no longer needed for transcription or verification, in accordance with the study's data management procedures and the participant information sheet. Transcripts used for analysis were pseudonymized before analysis, and direct identifiers were not retained in the analysis files.

3.6 File organization and naming

Study files were organized by data type and analysis stage, including consent materials, raw responses, transcripts, extracted scores, coding files, analytic memos, and supplementary artifacts. Participant-related files used pseudonymized participant identifiers rather than names. Consent materials were stored separately from response and analysis files.

3.7 Backup strategy

Study data were stored in a restricted-access University of Amsterdam research environment with institutional security and backup provisions. Local working copies, where used, were kept only as needed for analysis and were not used as the primary storage location.

3.8 File exchange with collaborators

Identifiable or raw participant data were not exchanged with collaborators outside the research team. Co-authors who participated in the study did not receive raw coded data for analysis or interpretation. Materials shared for manuscript development were anonymized, aggregated, or limited to study documentation where disclosure risk was low.

3.9 Pseudonymization and anonymization

Transcripts, written responses, extracted scores, and analytic files were pseudonymized before analysis using participant identifiers. Findings were anonymized in reporting by using broad role groupings and anonymized excerpts. Because the project setting is small and contextually identifiable, raw interview and questionnaire data are not publicly shared.

3.10 Analysis files

Pseudonymized data were used for qualitative coding, CRU score extraction, descriptive summaries, memoing, secondary-coder review, and RACI-based artifact development. Co-author participants were not involved in coding, data analysis, or interpretation of empirical findings.

3.11 External data

UNHCR Microdata Library data used for the hypothetical fairness risk scenario were accessed and analyzed under the applicable Terms of Use[1] and only for the approved scientific research purposes of the study. The external data were used only to construct the hypothetical scenario; no licensed microdata are reproduced, no respondent-level information is reported, no attempt was made to identify individuals, families, organizations, or communities, and the data were not used for operational or administrative decision-making. The external data were not linked to participant responses.

3.12 Data sharing

Raw interview and questionnaire data are not publicly shared because the sample is small and the project context creates a risk of indirect identification. Materials that can be shared as supplementary documentation include the questionnaire, interview protocol, coding protocol, codebook structure, RACI matrix, and role-specific guidance artifact, where disclosure risk is low.

3.13 Retention and deletion

Interview recordings and raw response materials will be deleted after project completion in accordance with the participant information sheet. Aggregated materials, pseudonymized analysis outputs, and manuscript-supporting documentation may be retained where required for transparency, auditability, or PhD thesis documentation, provided they do not include direct identifiers.

[1] `https://microdata.unhcr.org/index.php/terms-of-use`
