# Supplementary material for: Translating algorithmic fairness into health practice: formative evaluation of the fairness-to-action framework
Source: Front Artif Intell. 2026 Sep 21;9:1911214. doi: 10.3389/frai.2026.1911214 (PMC13635205; doi:10.3389/frai.2026.1911214)
Supplement: Supplementary file 3 [file Data_Sheet_3.pdf]

Interview Information Sheet

Evaluation of the Fairness-to-Action (F2A) Framework in Digital Health

Purpose of the Study. This study, conducted at the University of Amsterdam, examines how **algorithmic fairness** is understood and practiced in digital health initiatives such as *HealthEmove*. The goal is to explore whether the **Fairness-to-Action (F2A)** framework accurately reflects how fairness-related knowledge moves from ideas into action within real projects. The interview helps assess whether the framework's prompts are **clear, relevant, and usable** across different professional roles, capturing both conceptual understanding and practical application.

Why You Have Been Invited. You have been invited because your role provides valuable insight into how decisions are made, what challenges arise, and what helps or hinders fairness in digital health work. No technical expertise is required; your professional perspective is what matters most.

What Participation Involves. Participation takes the form of a written interview completed through a questionnaire. If you prefer, the interview can also be conducted in person or online through a scheduled video call. Questions focus on your experience with fairness, collaboration, and decision-making in the project. No preparation is needed; please respond based on your current understanding and as fully as you wish.

Voluntary Participation and Withdrawal. Your participation is entirely voluntary. You may skip any question or stop at any time without explanation or consequence.

Confidentiality and Data Protection. Your responses will not include any identifying information. All data will be kept securely on University of Amsterdam systems and used only for research purposes related to this study. Interview data will be deleted after the project concludes.

Consent. By submitting your responses, or by signing below if completing online, you confirm that you:

- have read and understood this information sheet;
- agree to participate voluntarily;
- consent to anonymized quotations being used in research outputs.

Researcher: Sara Altamirano, PhD Candidate, Informatics Institute, University of Amsterdam.
Email: s.e.altamirano@uva.nl

Participant Name:

Signature or initials: **Date:**

Questionnaire: Fairness-to-Action (F2A) Framework Evaluation in Digital Health

Introduction for Participants

Thank you for taking part in this study. We increasingly rely on artificial intelligence (AI)-driven systems across many areas of society. Institutions, researchers, and communities want these systems to be fair and transparent in practice. However, research shows that algorithmic fairness is often misunderstood, which can create gaps between fairness goals and what happens in real projects. This study explores where such gaps arise, and your answers will help us understand how fairness knowledge is used in practice.

There is also a growing body of work on how to design, assess, and govern fair and accountable AI systems. This area is complex and still evolving. In this study, we examine how these ideas apply in a practical setting and how they relate to the HealthEmove project. Your insights will help identify which fairness concepts are clear, which are challenging, and which are most relevant for your role.

The following questions ask about your understanding of *algorithmic fairness* in the context of the *HealthEmove* project. We are interested in how fairness is currently approached and how these ideas might apply once AI automation is introduced.

What do we mean by *algorithmic fairness*?
Algorithmic fairness means that AI models do not systematically disadvantage some groups compared with others. For example, if an AI tool gave people from one language group lower priority for follow-up than others with the same needs, this would be unfair.

What is this questionnaire evaluating?
The F2A framework describes different points where fairness knowledge can move from awareness into action (see Figure 1). These points are organized into three areas: What fairness knowledge exists, How it is used and coordinated in practice, and Why gaps in use persist. In this interview we also ask about the clarity, relevance, and usability of these points for the HealthEmove project.

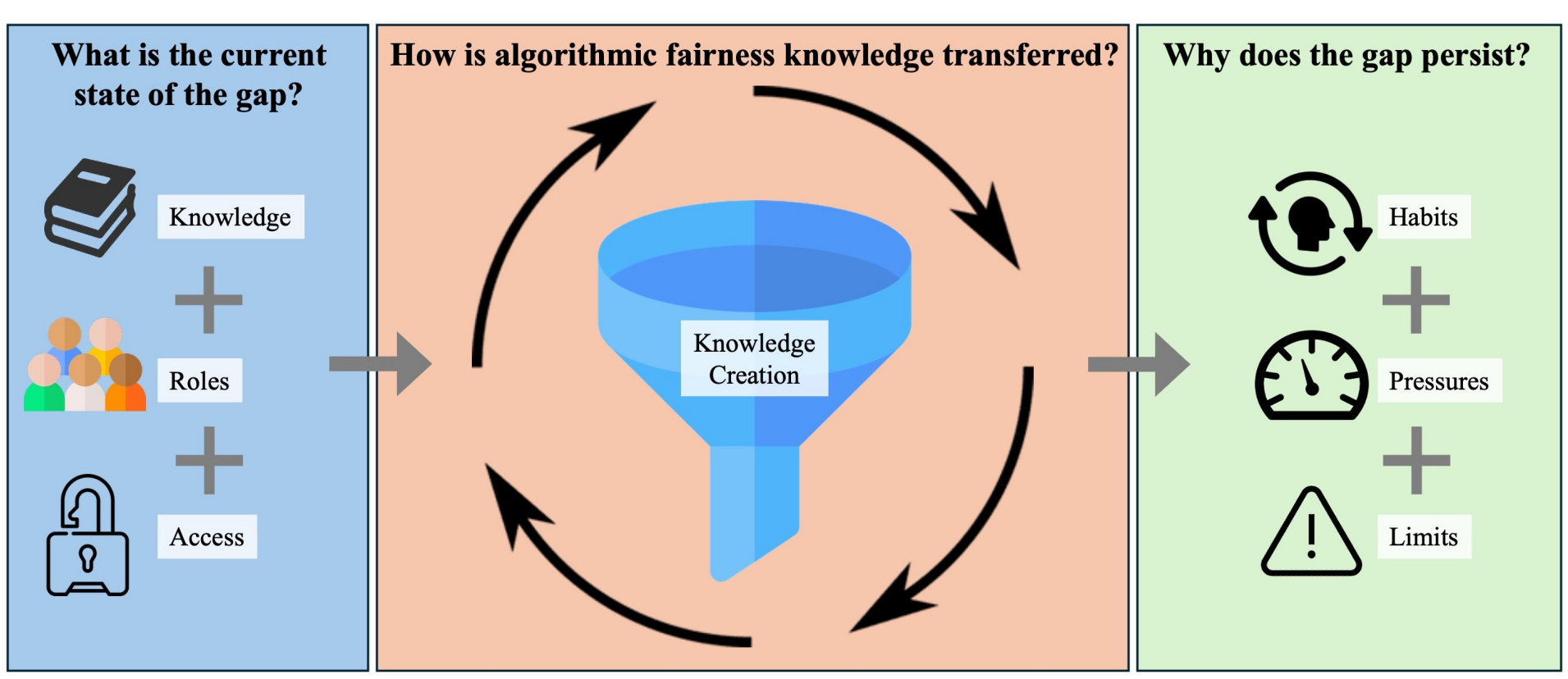

Figure 1: Simplified visual summary of the Fairness-to-Action (F2A) framework. The left panel shows **What** defines the current fairness gap, including knowledge, roles, and access. The center panel shows **How** fairness knowledge moves into practice. The right panel shows **Why** gaps persist, including habits, pressures, and limits. See Appendix A for the full F2A figure with the twenty-two points marked.

Section 1. Exploring Fairness-to-Action (F2A) in Practice

Each question below matches one point in the F2A framework.

- Please answer in your own words; short answers are fine.
- If something is not yet in place in HealthEmove, you can answer based on what you think would be realistic or appropriate.
- You may write "not involved" or "I don't know" if you believe a question does not apply to your role.

After each question you will be asked to rate:

- **Clarity:** how easy the item is to understand.
- **Relevance:** how well the item fits your context.
- **Usability:** how feasible it would be to apply this idea in practice.

Please use the same scale for all ratings: **1: very low, 2: low, 3: moderate, 4: high, 5: very high**.

WHAT. What algorithmic fairness knowledge exists?

1. What kinds of knowledge or guidance do you currently have, or think you would need, to make digital or data systems algorithmically fair and equitable for different users?

   Clarity (1–5): ☐ Relevance (1–5): ☐ Usability (1–5): ☐

2. Who in the project currently has, or should have, the main responsibility for algorithmic fairness knowledge or expertise?

   Clarity (1–5): ☐ Relevance (1–5): ☐ Usability (1–5): ☐

3. From whom do you or your team usually get information or advice about algorithmic fairness? (If this does not happen now, where do you think it should come from?)

   Clarity (1–5): ☐ Relevance (1–5): ☐ Usability (1–5): ☐

Scale for Clarity, Relevance, and Usability ratings: 1: very low, 2: low, 3: moderate, 4: high, 5: very high.

4. How do team members access information or support about algorithmic fairness when they need it? (If this is not yet in place, how could it be made available?)

Clarity (1–5): Relevance (1–5): Usability (1–5):

5. Has any knowledge about algorithmic fairness ever led, or could it realistically lead, to changes in how the project is designed or managed? (If not yet, what would help make this happen?)

Clarity (1–5): Relevance (1–5): Usability (1–5):

HOW. How is algorithmic fairness knowledge transferred?

6. How does HealthEmove generate or capture new lessons related to algorithmic fairness or inclusion? (If this is not yet happening, how might it be done?)

Clarity (1–5): Relevance (1–5): Usability (1–5):

7. How does your team bring together what is already known about algorithmic fairness from different partners or sources? (If this is not yet happening, how might it be done?)

Clarity (1–5): Relevance (1–5): Usability (1–5):

8. Are there any practical tools, templates, or examples that help you apply algorithmic fairness principles in daily work? (If not, what kind of resources would be useful?)

Clarity (1–5): Relevance (1–5): Usability (1–5):

Scale for Clarity, Relevance, and Usability ratings: 1: very low, 2: low, 3: moderate, 4: high, 5: very high.

9. How do you or others identify when algorithmic fairness might become an issue in your activities or decisions? (If this is not yet happening, how might it be done?)

Clarity (1–5): Relevance (1–5): Usability (1–5):

10. How do you decide which approaches or methods to use when considering algorithmic fairness? (If none are used, what would make selection easier?)

Clarity (1–5): Relevance (1–5): Usability (1–5):

11. How are ideas about algorithmic fairness adjusted to fit HealthEmove's specific goals, user groups, and context? (If not yet done, what would be important to adapt?)

Clarity (1–5): Relevance (1–5): Usability (1–5):

12. What barriers or challenges make it difficult to act on algorithmic fairness goals in your work?

Clarity (1–5): Relevance (1–5): Usability (1–5):

13. What kinds of concrete actions, if any, are being taken, or could realistically be taken, to make HealthEmove's data or systems more algorithmically fair?

Clarity (1–5): Relevance (1–5): Usability (1–5):

Scale for Clarity, Relevance, and Usability ratings: 1: very low, 2: low, 3: moderate, 4: high, 5: very high.

14. Is there any process for tracking or reviewing algorithmic fairness issues over time? (If not, what kind of simple tracking could work in practice?)

Clarity (1–5): Relevance (1–5): Usability (1–5):

15. Are there ways, formal or informal, to learn from previous experiences with algorithmic fairness or inclusion? (If not, what might enable that learning?)

Clarity (1–5): Relevance (1–5): Usability (1–5):

16. How could algorithmic fairness practices continue if key people or projects change? (What would help make them part of ongoing routines?)

Clarity (1–5): Relevance (1–5): Usability (1–5):

WHY. Why do algorithmic fairness efforts succeed or stall?

17. When you think of algorithmic fairness, do you see it mainly as a technical matter (for example, data quality or model accuracy) or also as an ethical and social responsibility? Why?

Clarity (1–5): Relevance (1–5): Usability (1–5):

18. Is algorithmic fairness something that people mostly discuss, or something that is also acted upon? What explains this balance?

Clarity (1–5): Relevance (1–5): Usability (1–5):

Scale for Clarity, Relevance, and Usability ratings: 1: very low, 2: low, 3: moderate, 4: high, 5: very high.

19. Is algorithmic fairness work usually about reporting requirements, or about learning and improvement? What would make it more meaningful?

Clarity (1–5): Relevance (1–5): Usability (1–5):

20. Do concerns such as performance targets or reputation ever discourage people from addressing algorithmic fairness issues openly? If so, how could that be reduced?

Clarity (1–5): Relevance (1–5): Usability (1–5):

21. Are goals such as accuracy or efficiency sometimes prioritized over algorithmic fairness? How could both be balanced more effectively?

Clarity (1–5): Relevance (1–5): Usability (1–5):

22. Do differences between disciplines or partner organizations make it harder to collaborate on algorithmic fairness? If yes, what would improve cooperation?

Clarity (1–5): Relevance (1–5): Usability (1–5):

Additional comments:

Section 2. Reflection and Feedback on the F2A Framework

This section asks about your overall impressions of the F2A framework, including how well its structure fits your experience, how complete it feels for capturing fairness issues, and how useful it would be in practice. For each item, please enter a single number from 1 to 5 in the box provided, using the same scale as before: 1: very low, 2: low, 3: moderate, 4: high, 5: very high.

Scale for Clarity, Relevance, and Usability ratings: 1: very low, 2: low, 3: moderate, 4: high, 5: very high.

A. Fit of the Structure

1. How clear is the overall three-part structure of the F2A framework for your work?

   1: very low, 2: low, 3: moderate, 4: high, 5: very high.

   Your rating (1–5):

2. How well does the F2A structure reflect how fairness actually works in HealthEmove?

   1: very low, 2: low, 3: moderate, 4: high, 5: very high.

   Your rating (1–5):

B. Completeness

3. How complete does the F2A framework feel for capturing fairness issues in HealthEmove?

   1: very low, 2: low, 3: moderate, 4: high, 5: very high.

   Your rating (1–5):

4. To what extent does F2A cover the main fairness processes relevant to your team?

   1: very low, 2: low, 3: moderate, 4: high, 5: very high.

   Your rating (1–5):

C. Practical Usefulness

5. How useful would F2A be for guiding fairness-related decisions in HealthEmove?

   1: very low, 2: low, 3: moderate, 4: high, 5: very high.

   Your rating (1–5):

6. How feasible would it be for your team to apply F2A in practice?

   1: very low, 2: low, 3: moderate, 4: high, 5: very high.

   Your rating (1–5):

Additional comments:

Section 3. Scenario Reflection

This section asks how you would interpret and respond to a hypothetical algorithmic fairness issue. For each item, please enter a single number from 1 to 5 in the box provided, using the same scale as before: 1: very low, 2: low, 3: moderate, 4: high, 5: very high.

Fairness Scenario. HealthEmove has an AI feature that predicts whether an uploaded medical document contains a chronic health condition. During routine monitoring, the team found that the model is about 21 percent more accurate for women aged 60+ than for other adult users, meaning it is more likely to miss chronic conditions in documents from other adults. *Note: real anonymized data from the United Nations High Commissioner for Refugees (UNHCR) Multi-Sector Needs Assessment (MSNA) 2023 was used to train a logistic regression model for this scenario.*

1. How serious is this fairness risk?

   1: very low, 2: low, 3: moderate, 4: high, 5: very high.

   Your rating (1–5): [ ]

2. How confident are you in the team identifying and interpreting this fairness issue?

   1: very low, 2: low, 3: moderate, 4: high, 5: very high.

   Your rating (1–5): [ ]

3. How important is it for the team to take action to address this issue?

   1: very low, 2: low, 3: moderate, 4: high, 5: very high.

   Your rating (1–5): [ ]

4. How strongly do you think this issue requires coordination between different partners or roles?

   1: very low, 2: low, 3: moderate, 4: high, 5: very high.

   Your rating (1–5): [ ]

5. How important is it to address this issue to ensure equitable support for users?

   1: very low, 2: low, 3: moderate, 4: high, 5: very high.

   Your rating (1–5): [ ]

6. How likely is this fairness issue to affect user trust or acceptance of HealthEmove?

   1: very low, 2: low, 3: moderate, 4: high, 5: very high.

   Your rating (1–5): [ ]

Additional comments:

Thank you for completing the questionnaire. If you have a moment to share any additional comments, please add them in Appendix B on the next page.

Appendix A. Full visual summary of the Fairness-to-Action (F2A) framework.

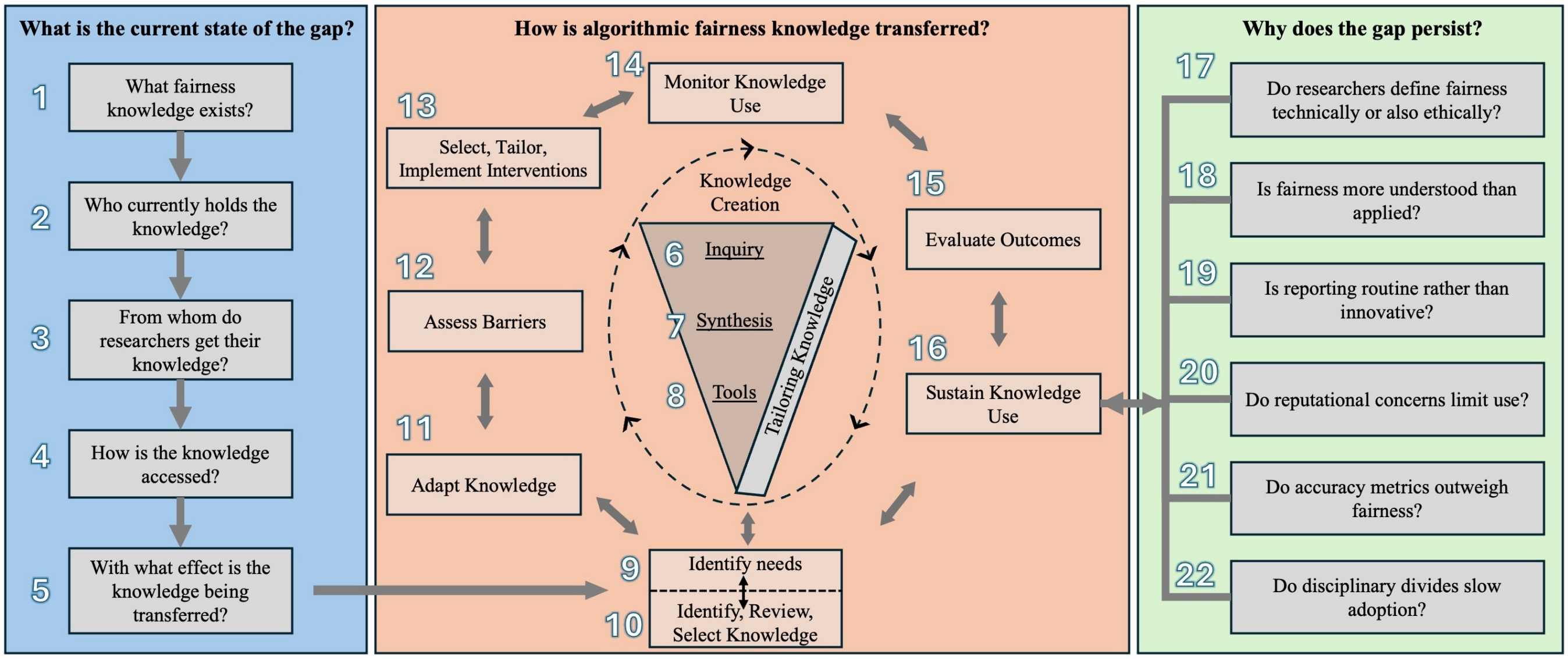

Figure 2: Full visual summary of the Fairness-to-Action (F2A) framework. The left section shows how fairness knowledge flows in a project, including what exists, who holds it, how it is accessed, and whether it leads to change. The middle section shows how this knowledge moves into practice through identification, creation, adaptation, sharing, implementation, monitoring, and sustainment. The right section shows why gaps continue, such as when fairness is treated narrowly, applied inconsistently, or shaped by organizational pressures or disciplinary divides. Together, the twenty-two items represent the full path from fairness ideas to fairness action.

Appendix B. Feedback on Questionnaire Design

Thank you for completing this questionnaire. Please provide brief feedback on the points below.

1. How clear did the questions feel overall?

   1: very low clarity, 2: low, 3: moderate, 4: high, 5: very high clarity.

   Your rating (1–5):

   Comment (optional):

2. How easy were the instructions to understand and follow?

   1: very low ease, 2: low, 3: moderate, 4: high, 5: very high ease.

   Your rating (1–5):

   Comment (optional):

3. How appropriate did the overall length and workload feel?

   1: very low appropriateness, 2: low, 3: moderate, 4: high, 5: very high appropriateness.

   Your rating (1–5):

   Comment (optional):

4. How realistic and answerable did the open-ended items feel for your role?

   1: very low realism, 2: low, 3: moderate, 4: high, 5: very high realism.

   Your rating (1–5):

   Any items that felt unclear, redundant, or confusing?

   Comment:

5. How intuitive and usable did the Clarity/Relevance/Usability rating scales feel?

   1: very low usability, 2: low, 3: moderate, 4: high, 5: very high usability.

   Your rating (1–5):

   Comment:

6. How relevant and clearly phrased did the Section 2 rating items feel?

   1: very low relevance/clarity, 2: low, 3: moderate, 4: high, 5: very high relevance/clarity.

   Your rating (1–5):

   Comment:

7. How well did the fillable fields function (text boxes, spacing, layout)?

   1: very low functionality, 2: low, 3: moderate, 4: high, 5: very high functionality.

   Your rating (1–5):

   Comment:

8. Suggestions to improve clarity, flow, usability, or other aspects.

   Comment:

9. Approximately how long did the full questionnaire take?

   minutes
