# Supplementary material for: Translating algorithmic fairness into health practice: formative evaluation of the fairness-to-action framework
Source: Front Artif Intell. 2026 Sep 21;9:1911214. doi: 10.3389/frai.2026.1911214 (PMC13635205; doi:10.3389/frai.2026.1911214)
Supplement: Supplementary file 4 [file Data_Sheet_4.pdf]

Supplementary Material D: Interview Protocol

1 F2A FORMATIVE EVALUATION INTERVIEW PROTOCOL

Study Title: Translating Algorithmic Fairness into Health Practice: Formative Evaluation of the Fairness-to-Action Framework
Project Context: HealthEmove: Digital Health for All initiative

1.1 Purpose and Design

This protocol documents the design and implementation of the structured interview questionnaire used for formative evaluation of the Fairness-to-Action (F2A) framework in a digital-health project setting.

The goal is to assess whether F2A prompts are clear, relevant, and usable across professional roles, and whether they reflect how fairness-related knowledge moves from awareness into project practice.

Because the study did not evaluate a deployed AI system, questions focused on fairness awareness, readiness, organizational learning, and perceived enablers and barriers for applying algorithmic fairness in current or future project stages.

The protocol supports transparency and replicability of the data-collection process and consistent administration across participants.

1.2 Methodological Approach

- **Type:** Structured interview questionnaire, completed in written form or through optional synchronous interviews.
- **Delivery:** Written questionnaire or researcher-administered interview containing open-ended responses and short evaluative items.
- **Participants:** HealthEmove and DFD-facing stakeholders representing diverse organizational roles, including technical, managerial, advisory, governance, public health, user experience, communication, and project coordination roles.
- **Sampling:** Purposive, role-stratified sampling based on involvement in the design, development, governance, or implementation of HealthEmove.
- **Evaluation Focus:** Each F2A prompt was assessed using three attributes: Clarity, Relevance, and Usability (CRU).

Justification for Written and Interview-Based Format. Participants could complete the questionnaire in writing or through an online or in-person interview. This flexible format was selected to accommodate participant availability, support reflection, and allow stakeholders to answer in the mode most suitable for their role and schedule. The use of the same questionnaire across formats supported consistency in data collection.

1.3 Interview Objectives

1. To elicit participants' understanding and experiences of algorithmic fairness in relation to their roles.
2. To evaluate how F2A prompts align with current or anticipated practices in digital-health work.
3. To identify contextual factors that enable or hinder fairness translation across the three F2A dimensions: What, How, and Why.

4. To collect descriptive CRU ratings for each F2A prompt as a complementary formative assessment measure.

1.4 Operational Details

- **Format:** Written questionnaire, online interview, or in-person interview, depending on participant preference and availability.
- **Estimated Duration:** Approximately 30-45 minutes.
- **Response Type:** Open-ended text responses and 1-5 Likert-scale ratings.
- **Confidentiality:** Responses were pseudonymized prior to analysis and reported without linking findings to identifiable individuals.

1.5 Definition of Algorithmic Fairness

For the purposes of this study, **algorithmic fairness** refers to whether AI models or data-driven systems systematically disadvantage some groups compared with others. In practical terms, this means examining whether digital or automated systems may produce unequal treatment, unequal performance, or unequal access to benefits across user groups.

1.6 Interview Structure

Section	Content Focus	Time
0. Information Sheet	Participant rights, data handling, and consent	2 min
1. Exploring F2A in Practice	Open-ended questions exploring the 22 F2A prompts across the What, How, and Why dimensions, each followed by CRU ratings	25-30 min
2. Framework Reflection and Feedback	Participant views on the structure, completeness, practical usefulness, and feasibility of the F2A framework	5 min
3. Scenario-Based Evaluation	Reflection on the Hypothetical Fairness Risk Scenario based on a potential HealthEmove AI-enabled feature	5 min
4. Questionnaire Design Feedback	Feedback on clarity, instructions, workload, answerability, rating scales, layout, and completion time	3-5 min

1.7 Evaluation Dimensions

Each F2A prompt was evaluated on three aspects:

1. **Clarity:** How easy the prompt was to understand.
2. **Relevance:** How well the prompt fit the participant's role or context.
3. **Usability:** How feasible it would be to apply the idea in practice.

All CRU ratings used the same 1-5 scale: 1 = very low, 2 = low, 3 = moderate, 4 = high, and 5 = very high.

1.8 Data Management and Confidentiality

- Data were collected through written responses or researcher-administered interviews, depending on participant preference.
- Responses were pseudonymized prior to analysis and reported in aggregate or through anonymized excerpts.
- Interview recordings, where applicable, were stored separately from transcripts, written responses, and extracted scores.
- Data were stored in a restricted-access University of Amsterdam research environment.
- All procedures followed University of Amsterdam research integrity guidance and GDPR data-protection principles.

1.9 Analysis Overview

- **Qualitative:** Qualitative analysis followed a framework-guided, item-level qualitative content analysis approach. Parent codes were derived from the 22 F2A prompts across the What, How, and Why dimensions, while subcodes were developed inductively from interview content to capture recurring patterns within each prompt.
- **Quantitative:** Descriptive statistics were used for CRU scores (Clarity, Relevance, and Usability ratings for each F2A prompt) and for additional scored items in the framework assessment, scenario-based evaluation, and questionnaire design feedback sections. CRU scores were interpreted as pragmatic, self-reported indicators of prompt-level interpretability and practical fit; no psychometric validation or inferential testing was conducted.
- **Integration:** Qualitative responses and descriptive ratings were interpreted complementarily to identify how stakeholders understood the F2A prompts, how ratings varied across items, and where role-specific interpretation or practical fit required further contextualization.

Expected Output. The formative assessment and mapping of F2A prompts to stakeholder practice was intended to support refinement of role-specific guidance for fairness-related work in a digital-health project setting.
