# Supplementary material for: Translating algorithmic fairness into health practice: formative evaluation of the fairness-to-action framework
Source: Front Artif Intell. 2026 Sep 21;9:1911214. doi: 10.3389/frai.2026.1911214 (PMC13635205; doi:10.3389/frai.2026.1911214)
Supplement: Supplementary file 5 [file Data_Sheet_5.pdf]

Supplementary Material E

RACI Matrix for Fairness-to-Action Tasks

Project: Translating Algorithmic Fairness into Health Practice: Formative Evaluation of the Fairness-to-Action Framework

Purpose: This matrix supports the evaluation of the Fairness to Action (F2A) framework within the HealthEmove Digital Health for All initiative. It provides the task structure and assignment procedure used to map who is Responsible, Accountable, Consulted, and Informed (RACI) for each algorithmic fairness task across the project.

Objective: To align HealthEmove stakeholders on roles and responsibilities for algorithmic fairness design, implementation, and governance, and to examine how fairness knowledge moves into action in a real organizational setting.

RACI definitions and assignment rules

- **R Responsible (Executor):** Executes the task and produces concrete outputs related to fairness work. Assign R if the role performs the task directly in practice, the role has time allocated for it, and the role would be responsible for the task being executed or not.
- **A Accountable (Decision authority):** Holds final decision authority and is accountable for outcomes and prioritization. Exactly one A per task. Assign A if the role can make/block decisions, can allocate resources or people, or the role carries institutional risk.
- **C Consulted:** Provides input, expertise, or feedback before or during task execution. The "Consulted" role is frequently assigned to senior employees, subject matter experts (SMEs), or stakeholders whose expertise is needed before a decision is finalized. Assign C if the role provides information, analysis, interpretation, or warning signals.
- **I Informed:** Is kept informed for alignment and transparency, without direct involvement in execution or decisions. Assign I if the role needs to know what happened for continuity, reporting, or learning; but does not act on it. The role does not execute the task, does not provide input that shapes the task, and only needs awareness of the outcome. Use sparingly.

Note: A correct matrix will show many blanks or Cs, very few Rs, one A per task, and responsibility increasing clearly as you move up roles.

Instructions for completing the original matrix

1. Review the Roles sheet to confirm the list of participants and role names.
2. Open the RACI Matrix sheet and read the instructions at the top, as well as the task definitions and examples.
3. For each task, assign R, A, C, or I based on how the project operates in practice.
4. Use brief notes only where clarification is needed.
5. Only edit yellow cells.

- For each task (row), enter only one letter per cell: R, A, C, or I. Do not enter other letters or symbols, and do not enter multiple letters in one cell.
- Ensure exactly one A is assigned per task; R, C, and I can be assigned to multiple roles per task.
- Not all roles need to be assigned to every task. Leave cells blank when a role is not involved.
- Base assignments on how HealthEmove operates in practice at the moment. If you disagree or are uncertain, add a brief note in the Notes column rather than forcing an assignment.

Notes

A) Interpretation of the RACI matrix: The RACI matrix reflects expected or current practice and is used for research and formative evaluation purposes. It is not a prescriptive governance model. Roles may confirm, revise, or challenge assignments based on actual project experience. This RACI mapping is conducted at the level of project roles, not individual people.

B) Data handling and privacy: No personal identifiers are included in this supplementary version. The completed working matrix was limited to invited participants and is not included here to reduce attribution risk in a small project setting. Inputs represented professional role perspectives and perceptions.

RACI matrix

Table E1. RACI matrix for F2A-derived fairness tasks. The task columns reproduce the task labels, short forms, long forms, and concrete examples used for responsibility mapping. Role columns are included as placeholders for R, A, C, or I assignments where applicable. Blank cells indicate that no completed role assignment is shown in this supplementary version. Role A and Role B are included as additional empty columns for project-specific role expansion.

No.	Item Label	F2A Task (short form)	F2A Task (long form)	Concrete example (fully specified)	Role A	Role B	Notes
0	EXAMPLE_ONLY	Raise fairness implications in key decision moments	Ensure that potential fairness impacts on different user groups are explicitly considered before major project decisions are finalized	During a decision meeting, potential negative impacts on certain user groups are explicitly raised, discussed, and taken into account before a final decision is made.			
1	WHAT_Knowledge_Availability	Identify available fairness knowledge	Identify and document what fairness-related knowledge, guidance, or evidence is available to support fair use of data or algorithms in the project	Creating and maintaining a short document that explains what fairness means for this project, lists known risks for different user groups, and names which guidelines or rules should be used when decisions about data or technology are made			
2	WHAT_Responsibility_Holders	Assign ownership of fairness expertise	Define who is responsible for holding, maintaining, and updating expertise on algorithmic fairness within the project	Officially assigning one role or person who is expected to answer fairness questions, be invited to relevant meetings, raise fairness concerns when they are missing, and check that raised concerns lead to follow-up actions or decisions			
3	WHAT_Knowledge_Sources	Identify sources of fairness advice	Identify the main internal and external sources from which the team receives advice or information about algorithmic fairness	Actively seeking advice from clearly identified sources such as a named legal advisor, a health expert, or a partner organization when fairness questions arise, rather than relying on informal assumptions			
4	WHAT_Knowledge_Access	Enable access to fairness support	Ensure that team members can easily access fairness-related information, guidance, or support when they need it	Making fairness information easy to find by placing it in a shared folder or guide, telling new team members where to find it, and reminding the team of its existence when relevant decisions are made			
5	WHAT_Knowledge_Effects	Apply fairness knowledge to decisions	Ensure that fairness knowledge can realistically influence project design choices, management decisions, or priorities	Changing a design choice, project rule, or priority after recognizing that an earlier plan could disadvantage certain users, and documenting why the change was made			
6	HOW_Inquiry_Process	Generate new fairness insights	Generate or capture new lessons about fairness or inclusion through project activities, evaluations, or reflection	Writing down lessons from concrete events such as a pilot, user complaint, review meeting, or internal discussion that revealed a fairness or inclusion issue			
7	HOW_Knowledge_Synthesis	Synthesize fairness knowledge	Bring together and consolidate fairness-related knowledge from different partners, disciplines, or sources	Producing a short written summary that combines input from different roles or partners and explains what the fairness issue is, why it matters, and what options exist			
8	HOW_Fairness_Tools	Provide practical fairness tools	Develop, select, or use practical tools, templates, or examples that help apply fairness principles in daily work	Using simple aids such as a checklist before decisions, example cases during discussions, or short written guidance that helps people think about fairness in their daily tasks			
9	HOW_Needs_Identification	Identify emerging fairness issues	Identify situations or decisions in which algorithmic fairness may become a concern or risk	Actively watching for warning signs such as certain users being excluded, struggling to use a feature, or being treated differently, and explicitly flagging these moments for discussion			

Continued on next page

Table E1 continued. RACI matrix for F2A-derived fairness tasks.

No.	Item Label	F2A Task (short form)	F2A Task (long form)	Concrete example (fully specified)	Role A	Role B	Notes
10	`HOW_Knowledge_Selection`	Select appropriate fairness methods	Decide which fairness approaches, methods, or frameworks are most appropriate to use in a given situation	Making a deliberate choice about how to respond to a fairness issue, for example deciding whether to change a process, collect additional information, delay a decision, or involve another expert			
11	`HOW_Knowledge_Adaptation`	Adapt fairness approaches to context	Adapt fairness approaches so they fit the project's goals, user groups, context, and constraints	Adjusting general fairness ideas so they fit real constraints, such as limited time, available data, language barriers, or the specific needs of the target users			
12	`HOW_Barrier_Assessment`	Assess barriers to fairness action	Identify organizational, technical, or resource-related barriers that make it difficult to act on fairness goals	Writing down concrete reasons why fairness action is difficult, such as unclear authority, lack of time, missing expertise, or conflicting priorities, instead of leaving them implicit			
13	`HOW_Intervention_Implementation`	Implement fairness actions	Plan and carry out concrete actions to improve fairness in data practices, system design, or implementation	Carrying out specific actions such as changing how information is collected, modifying a decision rule, updating documentation, or altering a workflow to reduce unfair outcomes			
14	`HOW_Use_Monitoring`	Monitor fairness over time	Track and periodically review fairness-related issues and actions over time	Keeping a simple log or recurring agenda item where fairness issues are recorded and revisited to see whether they have been addressed			
15	`HOW_Outcome_Evaluation`	Evaluate fairness outcomes	Learn from previous fairness-related experiences and assess whether actions led to meaningful improvements	Holding a brief review or reflection session to discuss what was done about fairness, what worked, what failed, and what should change next time			
16	`HOW_Use_Sustainment`	Sustain fairness practices	Embed fairness practices into routines and processes so they continue despite staff or project changes	Adding fairness checks to routine practices such as standard meetings, approval steps, onboarding materials, or recurring reviews so they do not depend on individual initiative			
17	`WHY_Fairness_Framing`	Frame fairness beyond technical terms	Ensure that fairness is understood and addressed as both a technical issue and an ethical or social responsibility	During a decision about changing a system or process, someone raises how the change could affect different groups of users, and this is discussed alongside efficiency or performance considerations.			
18	`WHY_Action_Gap`	Translate fairness discussion into action	Ensure that discussions about fairness are translated into concrete actions and changes in practice	Turning a fairness concern raised in a meeting into a concrete decision or action, such as changing a feature, adjusting a rule, or assigning follow-up responsibility			
19	`WHY_Routine_Reporting`	Use reporting for learning and improvement	Use fairness reporting activities to support learning, reflection, and improvement rather than only compliance	Using fairness reports or summaries to decide next steps, improvements, or changes, rather than only submitting them to satisfy a requirement			
20	`WHY_Reputational_Concerns`	Address reputational and performance pressures	Reduce performance or reputational pressures that discourage open discussion of fairness issues	Making it acceptable to raise fairness concerns even if this causes delays, extra work, or uncomfortable discussions, and showing through leadership behavior that this is supported			
21	`WHY_Metric_Dominance`	Balance performance and fairness goals	Balance goals such as accuracy or efficiency with fairness considerations when making decisions	Explicitly acknowledging and discussing situations where improving speed or efficiency may negatively affect some users, and deciding how to balance these goals			
22	`WHY_Disciplinary_Divides`	Support cross-disciplinary collaboration on fairness	Support effective collaboration across disciplines or partner organizations on fairness-related work	Setting up regular interaction across roles or organizations, such as joint meetings or shared decision moments, where fairness issues are discussed together rather than in isolation			

Prospective guidance for local F2A implementation and maintenance

This post-study guidance specifies recommended steps for adapting and maintaining the F2A responsibility-mapping procedure in a local setting. It was not presented to participants or evaluated as a transferable implementation protocol. The guidance draws on implementation-science recommendations to specify actors, actions, targets, and timing, and to document what was adapted, why, when, and by whom (Proctor et al., 2013; Miller et al., 2021). It also reflects evidence that Responsible AI procedures require alignment with organizational workflows, system lifecycle stages, and locally situated authority (Madaio et al., 2020; Widder and Nafus, 2023).

1. **Define the scope and facilitation process.** Specify the use case, system boundary, lifecycle stage, participating roles, facilitator, maintenance owner, and authority for resolving disputed assignments. For example, unresolved ownership of a monitoring task should be referred to a named governance body rather than left unassigned.
2. **Remap responsibilities locally.** Reconstruct the RACI mapping according to local roles, dependencies, workflows, and decision authority rather than transferring assignments from another setting. For example, approval responsibility should be assigned to the local role or governance body authorized to make the relevant decision.
3. **Document the instantiation.** For each retained F2A task, record its trigger, required action, expected output, role assignments, escalation route, and review timing. Specify whether escalation is event-based, such as an unresolved concern or absence of an owner, or threshold-based, such as a locally approved performance or incident threshold. Identify the Accountable role that decides the response and document disagreements, decisions, and their rationales. F2A does not prescribe universal numerical thresholds; these require definition and approval for the local use case and governance structure.
4. **Integrate the artifact with governance.** Connect task outputs to applicable approval, documentation, audit, incident-management, and reporting processes. For example, fairness evidence may be incorporated into an existing system-change review rather than maintained through a separate process.
5. **Maintain the mapping.** Review the artifact at a locally specified interval and when roles, decision authority, system functions, data, workflows, or governance requirements change. Each review should examine open escalations, documented follow-up, and whether assigned actions were completed. Version each revision, record its rationale and approval, and communicate it to affected roles.

References

Proctor, E. K., Powell, B. J., and McMillen, J. C. (2013). Implementation strategies: Recommendations for specifying and reporting. *Implementation Science, 8*, 139. `https://doi.org/10.1186/1748-5908-8-139`

Miller, C. J., Barnett, M. L., Baumann, A. A., Gutner, C. A., and Wiltsey-Stirman, S. (2021). The FRAME-IS: A framework for documenting modifications to implementation strategies in healthcare. *Implementation Science, 16*, 36. `https://doi.org/10.1186/s13012-021-01105-3`

Madaio, M. A., Stark, L., Vaughan, J. W., and Wallach, H. (2020). Co-designing checklists to understand organizational challenges and opportunities around fairness in AI. In *Proceedings of the 2020 CHI Conference on Human Factors in Computing Systems*, 1–14. `https://doi.org/10.1145/3313831.3376445`

Widder, D. G., and Nafus, D. (2023). Dislocated accountabilities in the "AI supply chain": Modularity and developers' notions of responsibility. *Big Data & Society, 10*(1). `https://doi.org/10.1177/20539517231177620`
