# Supplementary material for: Translating algorithmic fairness into health practice: formative evaluation of the fairness-to-action framework
Source: Front Artif Intell. 2026 Sep 21;9:1911214. doi: 10.3389/frai.2026.1911214 (PMC13635205; doi:10.3389/frai.2026.1911214)
Supplement: Supplementary file 6 [file Data_Sheet_6.pdf]

Algorithmic Fairness Recommendations and Checklist
Role: Junior Researcher (JR) for HealthEmove

The recommendations (Page 1) support continuous awareness and note-taking, while the checklist (Page 2) supports action at specific moments during project work.

Recommendations for daily work

When to use this guide: During meetings or daily project work, use this document when decisions, results, or questions arise about how Artificial Intelligence (AI) in HealthEmove may affect different user groups. For example, when discussing how the AI detects patterns in user data and whether it will work equally well for all age groups. **Algorithmic fairness** refers in this context to identifying whether an AI-supported feature within HealthEmove may affect some individuals or groups differently.

1. Keep a simple 'Fairness Knowledge Document' up to date

Maintain a shared file titled **Fairness Knowledge Document** in the project's Google Drive and keep it up to date. Add sources whenever they are mentioned or used in meetings, drafts, presentations, email exchanges, or supervision. Sources may include experts, guidelines, articles, examples, regulations, or internal notes.

For each source, record: title or short name, link or file path, where it was mentioned, reason for adding, and date.

If unsure, add the source and flag it for review by the Project Leader (PL).

2. Record fairness-related moments as they happen

Use the **Fairness Knowledge Document** to capture situations that may relate to how AI outputs, data, or decisions affect different user groups. Typical situations include when a decision refers to fairness, bias, inclusion, access, or unequal effects, when a dataset, model, AI feature, or automated output is discussed, when a user group may be affected differently, or when a concern is raised but no owner or next step is named.

For each entry, record: date, meeting, file, or activity, a short observation (1 to 2 sentences), potentially affected group(s), and any owner or follow-up person if named.

3. Use simple trigger questions when you are unsure

You do not need to be an expert in algorithmic fairness to use this tool. If you are unsure whether something is fairness-relevant, ask these questions:

- Could this affect some user groups differently from others?
- Is an important user group missing from the data, discussion, or example?
- Is someone making a claim about AI fairness, bias, or impact without evidence?

If the answer to any question is yes or unclear, record the situation in the **Fairness Knowledge Document**. If you remain unsure, consult the sources listed at the bottom of this page.

4. Escalate once, clearly, and record that you did so

If you notice a potential fairness issue, barrier, or unresolved concern, raise the issue with the PL or Research Assistant (RA). The message should state what you observed, who may be affected, where it occurred, and possible next step.

Possible next steps include adding the issue to an agenda, assigning an owner, reviewing a document, result, or decision, or clarifying what evidence exists.

After raising the issue, record in the **Fairness Knowledge Document** that it was escalated and to whom.

5. Write for handover and continuity

Assume someone else may need to continue your work. Use clear dates, meeting names, file names, links, and role labels so your notes can be understood by others.

When drafting text describing users, data, AI system behavior, or outcomes, include what is known, what is unclear, what data or evidence is missing, and what cannot yet be concluded. Keep the privacy of the users in mind.

Before leaving the role, share the location of the **Fairness Knowledge Document** with the PL.

Sources for further guidance

- **European Union (2024)**. *Artificial Intelligence Act.* **Use when:** checking legal requirements, obligations, and compliance for fair and trustworthy AI systems.
- **World Health Organization (2023)**. *Regulatory considerations on artificial intelligence for health.* **Use when:** assessing risk, equity, and safe use of AI in health contexts.
- **Raji et al. (2020)**. *Closing the AI accountability gap: Defining an end-to-end framework for internal algorithmic auditing.* **Use when:** documenting concerns, decisions, responsibilities, and follow-up actions.
- **Mitchell et al. (2019)**. *Model cards for model reporting.* **Use when:** documenting AI model use, limitations, and risks.
- **Barocas et al. (2023)**. *Fairness and machine learning: Limitations and opportunities.* **Use when:** understanding key concepts, trade-offs, and common fairness problems in AI systems.

Checklist

This checklist operationalizes selected tasks from **Fairness-to-Action (F2A)**, a knowledge translation framework used to examine what algorithmic fairness-related knowledge is available, how it is used in project work, and why it may not lead to action. We derived the tasks in the checklist based on the RACI (Responsible, Accountable, Consulted, Informed) matrix, and reflect the tasks assigned to the JR as 'R', while indicating relevant 'C' and 'I' roles as needed. The checklist structure is informed by existing reporting and assessment frameworks.

Instructions:

- Use this checklist during meetings, document preparation, data analysis discussions, and system design or implementation activities.
- When a situation described in the **Trigger** column occurs, complete the corresponding **Required Action** immediately. You may consult and inform the roles listed in the third and fourth columns.
- To support traceability and review, record all outputs in the **Fairness Knowledge Document** using short, factual entries.
- Each entry must include: what was observed, where it occurred, and any named next step or responsible role.
- Do not interpret, evaluate, or resolve issues unless explicitly instructed. Consult the Research Assistant (RA) or Project Leader (PL) when in doubt.

	**Trigger (F2A Task)**<br>When to act?	**Required Action**<br>What to do?	**Purpose**<br>Why does it matter?	**Consult**<br>Input	**Inform**<br>Notify
❑	**F2A-1 Knowledge Availability (R)**<br>A source is cited, shared, or referenced in a meeting, document, email, or presentation (e.g., law cited, guideline shared, expert advice given).	Add entry to **Fairness Knowledge Document**: date, source name, link/file, where it appeared, reason for adding.	Ensures sources are available for later review and decisions	CL, RAD, DS	SL, TPO
❑	**F2A-6 Inquiry Process (R)**<br>A situation occurs where outcomes differ across users or do not match expectations (e.g., model output differs by age group, unexpected model output, user complaint, mismatch between system output and user needs).	Add entry to **Fairness Knowledge Document**: what happened, where, affected group(s), named follow-up or owner (if any).	Ensures issues are recorded before they are forgotten or ignored	CL, RAD	SL, DS
❑	**F2A-7 Knowledge Synthesis (R)**<br>You are asked to combine input from multiple meetings, roles, or documents, or multiple inputs appear in the same discussion (e.g., combining meeting notes, partner input, draft findings).	Add synthesis to **Fairness Knowledge Document**: issue, affected group(s), key inputs, open questions, next step or owner.	Ensures inputs from different sources are captured in one place	CL, RAD	SL, DS
❑	**F2A-12 Barrier Assessment (R)**<br>Someone states that action cannot be taken due to a constraint (e.g., missing data, unclear responsibility, lack of time, conflicting priorities).	Add entry to **Fairness Knowledge Document**: barrier, who raised it, suggested next step (if any).	Ensures barriers are visible and can be addressed	IT, CL, SL	PL
❑	**F2A-15 Outcome Evaluation (R)**<br>A previous issue, decision, or action is reviewed or discussed again (e.g., follow-up discussion, revisiting earlier decision, checking if a change improved results).	Add entry to **Fairness Knowledge Document**: what was reviewed, observed outcome, next step or owner (if any).	Tracks whether actions lead to change	CL, SL	RA, IT
❑	**F2A-19 Routine Reporting (R)**<br>You are asked to write or update content that describes users, data, AI system behavior, or outcomes (e.g., writing report section, meeting notes, documenting results, preparing slides).	Write entry in **Fairness Knowledge Document**: known facts, supporting evidence, uncertainties, missing data, limits of conclusions.	Ensures transparent and consistent communication	CL, RAD	PD

This checklist reflects tasks assigned to the JR role within the project and does not cover all fairness responsibilities across roles.

Roles: PL: Project Leader; JR: Junior Researcher; RA: Research Assistant; IT: IT Lead; CL: Clinical Lead; RAD: Research Advisor; PD: Program Director; SL: Strategy Lead; DS: Data Scientist; TPO: Technical Product Owner; AIE: AI Engineer.

Glossary:

- *Algorithmic bias:* model predictions systematically benefit or disadvantage certain groups without justified reason.
- *Fairness (algorithmic fairness):* absence of systematic disadvantage for specific groups in data, models, or outcomes.
- *Artificial intelligence (AI):* data-driven models used to generate predictions, classifications, or decisions.
- *Prediction model:* model estimating the probability of an outcome based on input data.
- *Predictor:* measurable input used by a model (e.g., age, symptom, score).
- *Outcome:* result produced by a system, model, or decision.
- *Model evaluation:* assessing how well a model performs on data.
- *User group:* individuals who may be affected differently by the system.
- *Source:* input informing fairness understanding (e.g., guideline, study, expert input).
- *Barrier:* factor preventing action on a fairness issue (e.g., missing data, unclear responsibility).
- *Synthesis:* short summary combining multiple inputs into a structured overview.
- *Fairness Knowledge Document:* project file for recording sources, observations, and actions.

Some definitions adapted from Moons, K. G. M., et al. (2025). *PROBAST+AI: An updated quality, risk of bias, and applicability assessment tool for prediction models using regression or artificial intelligence methods. BMJ, 388*, e082505.

Appendix A. Fairness Knowledge Document Template

Use the section that matches the **F2A item** in the checklist. Record entries briefly and factually. This template is designed for the **Fairness Knowledge Document** referenced in the recommendations and checklist.

F2A-1 Knowledge Availability

A source is cited, shared, or referenced in a meeting, document, email, or presentation (e.g., law cited, guideline shared, expert advice given).

Date	Source name	Link / File	Where mentioned	Reason for adding

F2A-6 Inquiry Process

A situation occurs where outcomes differ across users or do not match expectations (e.g., model output differs by age group, unexpected model output, user complaint, mismatch between system output and user needs).

Date	What happened	Where	Affected group(s)	Owner / follow-up

F2A-7 Knowledge Synthesis

You are asked to combine input from multiple meetings, roles, or documents, or multiple inputs appear in the same discussion (e.g., combining meeting notes, partner input, draft findings).

Issue	Affected group(s)	Key inputs	Open questions	Next step / owner

F2A-12 Barrier Assessment

Someone states that action cannot be taken due to a constraint (e.g., missing data, unclear responsibility, lack of time, conflicting priorities).

Date	Barrier	Who raised it	Suggested next step

F2A-15 Outcome Evaluation

A previous issue, decision, or action is reviewed or discussed again (e.g., follow-up discussion, revisiting earlier decision, checking if a change improved results).

Date	What was reviewed	Observed outcome	Next step / owner

F2A-19 Routine Reporting

You are asked to write or update content that describes users, data, AI system behavior, or outcomes (e.g., writing report section, meeting notes, documenting results, preparing slides).

What to include:	Content
Known facts<br>Supporting evidence<br>Uncertainties<br>Missing data<br>Limits of conclusions	

Reminder: Use clear dates, meeting names, file names, links, and role labels. Keep entries brief. Do not include unnecessary personal data.

Appendix B. Evaluation of the Junior Researcher Instrument

This section evaluates the **Recommendations (Page 1)** and the **Checklist (Page 2)**. Please write one rating from 1 to 5 in each box.

Scale: 1 = very low, 2 = low, 3 = moderate, 4 = high, 5 = very high.
Clarity (C): how easy the component is to understand
Relevance (R): how well the component fits your role and tasks
Usability (U): how easy the component would be to use in practice

A. Recommendations (Page 1)

Component	C	R	U
Instructions and overall explanation			
Recommendation 1: Fairness Knowledge Document			
Recommendation 2: Recording situations			
Recommendation 3: Trigger questions			
Recommendation 4: Escalation guidance			
Recommendation 5: Handover and continuity			
Sources for further guidance			

Comments on the recommendations (optional):

B. Checklist (Page 2)

Component	C	R	U
Checklist instructions			
F2A-1 Knowledge Availability			
F2A-6 Inquiry Process			
F2A-7 Knowledge Synthesis			
F2A-12 Barrier Assessment			
F2A-15 Outcome Evaluation			
F2A-19 Routine Reporting			
Roles and glossary			

Comments on the checklist (optional):

C. Overall assessment

1. How clear is the overall instrument for your role? Rating:

2. How relevant is the instrument for your daily tasks and responsibilities? Rating:

3. How usable is the instrument in real project work? Rating:

4. How complete is the instrument for supporting fairness-related work in your role? Rating:

5. Would you be able to use this instrument without additional guidance? Rating:

6. Which parts of the instrument were unclear, redundant, or difficult to use?

7. What improvements would make this instrument easier or more useful to apply in practice?
