# Supplementary material for: Translating algorithmic fairness into health practice: formative evaluation of the fairness-to-action framework
Source: Front Artif Intell. 2026 Sep 21;9:1911214. doi: 10.3389/frai.2026.1911214 (PMC13635205; doi:10.3389/frai.2026.1911214)
Supplement: Supplementary file 8 [file Data_Sheet_8.pdf]

Supplementary Material H: Technical Characterization of the Hypothetical Fairness Risk Scenario

1 PURPOSE AND SCOPE

This supplementary file provides a post hoc technical characterization of the proxy-model setup underlying the accuracy-based cue in the participant-facing hypothetical fairness risk scenario. The scenario used accuracy as a common, readily interpretable monitoring cue for discussion among participants with differing technical and professional backgrounds. Accuracy was not treated as a fairness metric or standalone fairness determination. The expanded analyses reported here were conducted subsequently and were not presented to participants; the scenario-based findings are therefore interpreted in relation to the hypothetical risk as presented.

The envisioned feature would convert uploaded documents into structured variables on recent health problems, access to care and reported barriers, and age. The proxy model used related survey variables to approximate this post-extraction information environment and provide a shared basis for assessing a hypothetical monitoring risk. Its purpose was to support participants' assessment of that risk, rather than reproduce the document-extraction process or evaluate the feature's technical performance or fairness. Future scenario evaluations should incorporate probability-based discrimination and calibration assessments, uncertainty estimates, and intersectional analyses where appropriate.

2 TECHNICAL CHARACTERIZATION METHODS

The source data were anonymized microdata from the UNHCR *Multi-Sector Needs Assessment (MSNA) - 2023* regional dataset (catalog 1057). The binary outcome was self-reported chronic condition status. The participant-facing scenario used 5,274 respondents aged 18 years and older with non-missing outcome, age, recent-health-problem, and access-to-care values and included every field matching the access-barrier prefix in the barrier composite. The post hoc characterization additionally required non-missing sex, yielding 5,273 respondents, and excluded the prefer-not-to-answer field from the composite; its estimates therefore differ slightly from those presented in the scenario. In both analyses, the barrier predictor indicated whether at least one included field was affirmatively coded, and fields unobserved because of access-to-care skip logic were not counted as reported barriers. The characterization used the same adult restriction, subgroup definition, 70/30 train-test split, outcome stratification, random seed of 42, logistic-regression specification, and 0.50 classification threshold as the scenario analysis. Because the access-to-care item was observed only for respondents reporting a recent health problem, the analytic specification produced no variation in the recent-health-problem indicator; it consequently had no influence on fitted predictions. Because age contributed both to predictions and subgroup membership, the comparisons characterize model behavior in the specified subgroups and do not estimate causal effects of age or subgroup membership. Metrics were calculated on the resulting held-out test set. We report subgroup sizes, outcome prevalence, accuracy, predicted-positive rate, area under the

receiver operating characteristic curve (AUROC), false-negative and false-positive rates, Brier score, and the calibration-in-the-large gap (observed prevalence minus mean predicted probability).

Uncertainty was estimated using 2,000 nonparametric bootstrap resamples of held-out observations within each subgroup, with a bootstrap seed of 42. Reported 95% percentile confidence intervals are conditional on the fitted model. The analysis used Python 3.12.13, pandas 2.2.2, NumPy 2.0.2, and scikit-learn 1.6.1.

3 TECHNICAL CHARACTERIZATION RESULTS

Table S1. Post hoc technical characterization of the proxy model on the held-out test set. Values in parentheses are 95% bootstrap percentile confidence intervals. Differences are women aged 60 years and older minus other adults. Point differences were calculated from unrounded estimates. Difference confidence intervals were calculated directly from bootstrap distributions of between-group differences and therefore need not equal differences between the corresponding marginal confidence-interval endpoints.

Metric	Women aged 60+	Other adults	Difference
Model sample size	1,270	4,003	–
Held-out test sample size	376	1,206	–
Outcome prevalence	0.872 (0.838, 0.904)	0.555 (0.527, 0.583)	0.318 (0.271, 0.363)
Accuracy	0.872 (0.838, 0.904)	0.646 (0.619, 0.674)	0.226 (0.182, 0.268)
Predicted-positive rate	1.000 (1.000, 1.000)	0.703 (0.677, 0.730)	0.297 (0.270, 0.323)
AUROC	0.480 (0.422, 0.534)	0.667 (0.640, 0.697)	-0.187 (-0.254, -0.127)
False-negative rate	0.000 (0.000, 0.000)	0.185 (0.156, 0.217)	-0.185 (-0.217, -0.156)
False-positive rate	1.000 (1.000, 1.000)	0.564 (0.520, 0.605)	0.436 (0.395, 0.480)
Brier score	0.115 (0.093, 0.137)	0.224 (0.215, 0.233)	-0.109 (-0.132, -0.085)
Calibration-in-the-large gap	0.045 (0.011, 0.078)	-0.008 (-0.035, 0.019)	0.052 (0.008, 0.097)

At the 0.50 classification threshold, all women aged 60 years and older were classified as positive. This produced a false-negative rate of 0 and a false-positive rate of 1.000: no observed positive cases were missed, while all observed negative cases were classified as positive. Accuracy in this subgroup therefore equaled outcome prevalence. AUROC was 0.480 (95% CI: 0.422, 0.534), compared with 0.667 (95% CI: 0.640, 0.697) among other adults. These results indicate that subgroup prevalence and threshold-specific classification behavior contributed to the accuracy difference. In this study, the difference functioned as a hypothetical monitoring flag requiring further assessment, rather than as evidence of unfairness or superior groupwise discrimination.

The calibration-in-the-large gaps were 0.045 among women aged 60 years and older and -0.008 among other adults. The confidence interval for the between-group difference excluded zero (0.052; 95% CI: 0.008, 0.097), indicating a between-group difference in calibration-in-the-large in this held-out sample. The lower Brier score among women aged 60 years and older is not interpreted alone as evidence of better calibration because Brier scores are influenced by outcome prevalence. The focal comparison combined sex and age but was not a comprehensive intersectional audit.

Subgroup net benefit was not estimated because decision-curve analysis requires a specified clinical decision, a meaningful decision threshold, and the relative consequences of false-positive and false-negative decisions. These elements were not defined in this non-clinical hypothetical scenario, so a net-benefit estimate would not be interpretable.

4 ANALYSIS CODE

The code below uses an explicit analysis-mode setting to reproduce either the participant-facing scenario analysis or the post hoc characterization. It preserves the complete analytic sequence, model parameters, metric definitions, and bootstrap procedure. To comply with the conditions governing the licensed third-party microdata, it does not include the data file, respondent-level information, local file paths, source-variable identifiers, or source-category encodings. Researchers with authorized access can populate the marked configuration values using the data documentation supplied with their approved copy. No licensed microdata are reproduced by this code.

Listing 1. Redacted Python code for reproducing the participant-facing scenario analysis and post hoc proxy-model characterization with an authorized copy of the source data.

```python
import pandas as pd
import numpy as np
from sklearn.linear_model import LogisticRegression
from sklearn.metrics import (
    accuracy_score,
    brier_score_loss,
    confusion_matrix,
    roc_auc_score,
)
from sklearn.model_selection import train_test_split


# ------------------------------------------------------------
# 1. Authorized data and dataset-specific mappings
# ------------------------------------------------------------
# Replace only with values from the documentation accompanying
# an authorized copy of the licensed source data.
DATA_FILE = "<PATH_TO_AUTHORIZED_DATA>"

# Use "post_hoc" to reproduce Table S1.
# Use "participant_facing" to reproduce the original scenario cue.
ANALYSIS_MODE = "post_hoc"

if ANALYSIS_MODE not in {"participant_facing", "post_hoc"}:
    raise ValueError("Invalid ANALYSIS_MODE")

COL = {
    "outcome": "<OUTCOME_COLUMN>",
    "age": "<AGE_COLUMN>",
    "sex": "<SEX_COLUMN>",
    "recent_problem": "<RECENT_PROBLEM_COLUMN>",
    "obtained_care": "<OBTAINED_CARE_COLUMN>",
}
BARRIER_PREFIX = "<BARRIER_COLUMN_PREFIX>"
EXCLUDED_BARRIER_COLUMNS = {
    "<PREFER_NOT_TO_ANSWER_COLUMN>"
}

YES_VALUE = "<AFFIRMATIVE_CATEGORY>"
NO_VALUE = "<NEGATIVE_CATEGORY>"
FEMALE_VALUE = "<FEMALE_CATEGORY>"
```

```
ADULT_AGE_CATEGORIES = ["<ADULT_CATEGORY_1>", "<...>"]
OLDEST_AGE_CATEGORY = "<AGE_60_PLUS_CATEGORY>"
AGE_ORDER = {
    "<ADULT_CATEGORY_1>": 0,
    "<ADULT_CATEGORY_2>": 1,
    "<ADULT_CATEGORY_3>": 2,
    "<AGE_60_PLUS_CATEGORY>": 3,
}
BARRIER_PRESENT_VALUES = ["<BARRIER_PRESENT_ENCODING>"]


# ------------------------------------------------------------
# 2. Outcome, analytic population, and subgroup
# ------------------------------------------------------------
df = pd.read_csv(DATA_FILE)
df["outcome"] = df[COL["outcome"]].map(
    {YES_VALUE: 1, NO_VALUE: 0}
)
df = df[df[COL["age"]].isin(ADULT_AGE_CATEGORIES)].copy()
df = df[df["outcome"].notna()].copy()
if ANALYSIS_MODE == "post_hoc":
    df = df[df[COL["sex"]].notna()].copy()

df["female"] = (df[COL["sex"]] == FEMALE_VALUE).astype(int)
df["age_60plus"] = (
    df[COL["age"]] == OLDEST_AGE_CATEGORY
).astype(int)
df["female_60plus"] = (
    (df["female"] == 1) & (df["age_60plus"] == 1)
).astype(int)


# ------------------------------------------------------------
# 3. Predictors
# ------------------------------------------------------------
df["recent_problem"] = df[COL["recent_problem"]].map(
    {YES_VALUE: 1, NO_VALUE: 0}
)
df["obtained_care"] = df[COL["obtained_care"]].map(
    {YES_VALUE: 1, NO_VALUE: 0}
)

all_barrier_columns = [
    c for c in df.columns
    if c.startswith(BARRIER_PREFIX)
]

if ANALYSIS_MODE == "post_hoc":
    barrier_columns = [
        c for c in all_barrier_columns
        if c not in EXCLUDED_BARRIER_COLUMNS
    ]
else:
    barrier_columns = all_barrier_columns
```

```
barrier_flags = pd.DataFrame(index=df.index)
for column in barrier_columns:
    barrier_flags[column] = (
        df[column].isin(BARRIER_PRESENT_VALUES).astype(int)
    )
df["any_barrier"] = (
    barrier_flags.sum(axis=1) > 0
).astype(int)
df["age_code"] = df[COL["age"]].map(AGE_ORDER)

FEATURES = [
    "recent_problem",
    "obtained_care",
    "any_barrier",
    "age_code",
]
model_data = df.dropna(
    subset=FEATURES + ["female_60plus"]
).copy()

expected_n = 5273 if ANALYSIS_MODE == "post_hoc" else 5274
assert len(model_data) == expected_n
print("Analysis mode:", ANALYSIS_MODE)
print("Model sample size:", len(model_data))


# ------------------------------------------------------------
# 4. Model and held-out predictions
# ------------------------------------------------------------
X = model_data[FEATURES]
y = model_data["outcome"]
group = model_data["female_60plus"]

X_train, X_test, y_train, y_test, g_train, g_test = (
    train_test_split(
        X,
        y,
        group,
        test_size=0.30,
        random_state=42,
        stratify=y,
    )
)

model = LogisticRegression(max_iter=1000)
model.fit(X_train, y_train)
probability = model.predict_proba(X_test)[:, 1]
prediction = model.predict(X_test)

y_array = y_test.to_numpy()
g_array = g_test.to_numpy()
group_index = np.flatnonzero(g_array == 1)
other_index = np.flatnonzero(g_array == 0)
```

```

# ------------------------------------------------------------
# 5. Aggregate subgroup metrics
# ------------------------------------------------------------
def subgroup_metrics(index):
    observed = y_array[index]
    predicted_probability = probability[index]
    predicted_class = prediction[index]
    tn, fp, fn, tp = confusion_matrix(
        observed, predicted_class, labels=[0, 1]
    ).ravel()

    return {
        "n": len(observed),
        "prevalence": observed.mean(),
        "accuracy": accuracy_score(
            observed, predicted_class
        ),
        "predicted_positive_rate": predicted_class.mean(),
        "auroc": roc_auc_score(
            observed, predicted_probability
        ),
        "false_negative_rate": fn / (fn + tp),
        "false_positive_rate": fp / (fp + tn),
        "brier_score": brier_score_loss(
            observed, predicted_probability
        ),
        "calibration_gap": (
            observed.mean() - predicted_probability.mean()
        ),
    }


group_metrics = subgroup_metrics(group_index)
other_metrics = subgroup_metrics(other_index)


# ------------------------------------------------------------
# 6. Conditional test-set bootstrap confidence intervals
# ------------------------------------------------------------
METRICS = [
    "prevalence",
    "accuracy",
    "predicted_positive_rate",
    "auroc",
    "false_negative_rate",
    "false_positive_rate",
    "brier_score",
    "calibration_gap",
]

rng = np.random.default_rng(42)
n_bootstrap = 2000
bootstrap = {
```

```
    "group": {metric: [] for metric in METRICS},
    "other": {metric: [] for metric in METRICS},
    "difference": {metric: [] for metric in METRICS},
}

for _ in range(n_bootstrap):
    sampled_group = rng.choice(
        group_index, size=len(group_index), replace=True
    )
    sampled_other = rng.choice(
        other_index, size=len(other_index), replace=True
    )
    metric_group = subgroup_metrics(sampled_group)
    metric_other = subgroup_metrics(sampled_other)

    for metric in METRICS:
        bootstrap["group"][metric].append(
            metric_group[metric]
        )
        bootstrap["other"][metric].append(
            metric_other[metric]
        )
        bootstrap["difference"][metric].append(
            metric_group[metric] - metric_other[metric]
        )


def percentile_interval(values):
    return np.quantile(values, [0.025, 0.975])


# Aggregate results only; no respondent-level values are printed.
for metric in METRICS:
    group_ci = percentile_interval(
        bootstrap["group"][metric]
    )
    other_ci = percentile_interval(
        bootstrap["other"][metric]
    )
    difference_ci = percentile_interval(
        bootstrap["difference"][metric]
    )
    difference = group_metrics[metric] - other_metrics[metric]

    print(
        metric,
        group_metrics[metric],
        group_ci,
        other_metrics[metric],
        other_ci,
        difference,
        difference_ci,
    )
```

```
print("group_n", group_metrics["n"])
print("other_n", other_metrics["n"])
print(
    "model_sample_group_n",
    int(model_data["female_60plus"].sum()),
)
print(
    "model_sample_other_n",
    int((model_data["female_60plus"] == 0).sum()),
)
```

5 DATA-USE STATEMENT

The licensed microdata are not included in this supplementary file and may not be reconstructed from the reported aggregate results or redacted code. Researchers seeking to reproduce the analysis must obtain their own authorization through the UNHCR Microdata Library and comply with the terms applicable to their approved access. The source data were used only for the scientific research purposes of this study and were not linked to interview-participant data. UNHCR does not warrant in any way the accuracy of the data or information reproduced from the Microdata Library and may not be held liable for any loss caused by reliance on the accuracy or reliability thereof. The analyses, interpretations, and conclusions reported here are solely those of the authors.
