# Supplementary material for: Translating algorithmic fairness into health practice: formative evaluation of the fairness-to-action framework
Source: Front Artif Intell. 2026 Sep 21;9:1911214. doi: 10.3389/frai.2026.1911214 (PMC13635205; doi:10.3389/frai.2026.1911214)
Supplement: Supplementary file 9 [file Data_Sheet_9.pdf]

Supplementary Material I: Item-level CRU distributions

This table reports the complete item-level distributions of Clarity, Relevance, and Usability (CRU) ratings for the 22 Fairness-to-Action prompts. Ratings used a five-point Likert scale (1 = very low, 2 = low, 3 = moderate, 4 = high, 5 = very high). Frequencies are counts of valid responses. IQR denotes the interquartile range, reported as the 25th–75th percentile interval. For all item-scale combinations, $n = 11$ except Relevance for *Knowledge Adaptation* and all three scales for *Routine Reporting*, where $n = 10$.

Table 1: Item-level CRU summary statistics and score frequencies.

**F2A item**	**CRU scale**	n	**Mean**	**SD**	**Median**	**IQR**	**Score frequency**				
							**1**	**2**	**3**	**4**	**5**
What											
Knowledge Availability	Clarity	11	3.6	1.1	4	3–4.5	0	2	3	3	3
	Relevance	11	4.4	0.7	4	4–5	0	0	1	5	5
	Usability	11	4.1	0.9	4	4–5	0	1	1	5	4
Responsibility Holders	Clarity	11	4.7	0.5	5	4.5–5	0	0	0	3	8
	Relevance	11	4.4	0.9	5	4–5	0	1	0	4	6
	Usability	11	4.5	0.7	5	4–5	0	0	1	4	6
Knowledge Sources	Clarity	11	4.6	0.5	5	4–5	0	0	0	4	7
	Relevance	11	4.5	0.5	5	4–5	0	0	0	5	6
	Usability	11	4.0	0.8	4	3.5–4.5	0	0	3	5	3
Knowledge Access	Clarity	11	4.1	1.0	4	3.5–5	0	1	2	3	5
	Relevance	11	4.5	0.5	5	4–5	0	0	0	5	6
	Usability	11	4.2	0.8	4	4–5	0	0	2	5	4
Knowledge Effects	Clarity	11	4.4	0.7	4	4–5	0	0	1	5	5
	Relevance	11	4.5	0.5	5	4–5	0	0	0	5	6
	Usability	11	4.4	0.7	4	4–5	0	0	1	5	5
How											
Inquiry Process	Clarity	11	4.4	0.9	5	4–5	0	1	0	4	6
	Relevance	11	4.6	0.7	5	4.5–5	0	0	1	2	8
	Usability	11	4.2	0.9	4	3.5–5	0	0	3	3	5
Knowledge Synthesis	Clarity	11	4.5	0.5	5	4–5	0	0	0	5	6
	Relevance	11	4.4	1.0	5	4–5	0	1	1	2	7
	Usability	11	4.5	1.0	5	4.5–5	0	1	1	1	8
Fairness Tools	Clarity	11	4.5	0.8	5	4–5	0	0	2	2	7
	Relevance	11	4.4	0.9	5	3.5–5	0	0	3	1	7
	Usability	11	4.3	1.0	5	4–5	0	1	1	3	6
Needs Identification	Clarity	11	4.0	1.1	4	3–5	0	1	3	2	5
	Relevance	11	4.5	0.7	5	4–5	0	0	1	4	6
	Usability	11	4.1	0.8	4	3.5–5	0	0	3	4	4
Knowledge Selection	Clarity	11	3.9	0.8	4	3–4.5	0	0	4	4	3
	Relevance	11	4.2	1.1	5	3.5–5	0	1	2	2	6
	Usability	11	4.0	1.0	4	3.5–5	0	1	2	4	4
Knowledge Adaptation	Clarity	11	4.1	0.8	4	3.5–5	0	0	3	4	4
	Relevance	10	4.4	0.5	4	4–5	0	0	0	6	4
	Usability	11	4.2	0.8	4	4–5	0	0	2	5	4
Barrier Assessment	Clarity	11	4.5	0.5	5	4–5	0	0	0	5	6
	Relevance	11	4.5	0.7	5	4–5	0	0	1	3	7
	Usability	11	4.2	1.1	5	3.5–5	0	1	2	2	6
Intervention Implementation	Clarity	11	4.6	0.7	5	4.5–5	0	0	1	2	8
	Relevance	11	4.5	0.7	5	4–5	0	0	1	3	7
	Usability	11	4.2	0.9	4	4–5	0	1	0	6	4

Continued on next page

Table 1 continued

F2A item	CRU scale	n	Mean	SD	Median	IQR	Score frequency				
							**1**	**2**	**3**	**4**	**5**
Use Monitoring	Clarity	11	4.4	0.8	5	4–5	0	0	2	3	6
	Relevance	11	4.4	0.8	5	4–5	0	0	2	3	6
	Usability	11	4.2	1.0	4	4–5	0	1	1	4	5
Outcome Evaluation	Clarity	11	4.5	0.5	5	4–5	0	0	0	5	6
	Relevance	11	4.5	0.7	5	4–5	0	0	1	4	6
	Usability	11	4.3	0.6	4	4–5	0	0	1	6	4
Use Sustainment	Clarity	11	4.7	0.5	5	4.5–5	0	0	0	3	8
	Relevance	11	4.5	0.7	5	4–5	0	0	1	3	7
	Usability	11	4.4	1.0	5	4–5	0	1	1	2	7
Why											
Fairness Framing	Clarity	11	4.6	0.5	5	4–5	0	0	0	4	7
	Relevance	11	4.1	1.0	4	3.5–5	0	1	2	3	5
	Usability	11	4.5	0.7	5	4–5	0	0	1	4	6
Action Gap	Clarity	11	4.6	0.5	5	4–5	0	0	0	4	7
	Relevance	11	4.5	0.7	5	4–5	0	0	1	4	6
	Usability	11	4.0	1.1	4	3–5	0	1	3	2	5
Routine Reporting	Clarity	10	4.1	0.9	4	4–4.75	0	1	0	6	3
	Relevance	10	4.2	0.8	4	4–5	0	0	2	4	4
	Usability	10	4.3	0.7	4	4–5	0	0	1	5	4
Reputational Concerns	Clarity	11	4.6	0.5	5	4–5	0	0	0	4	7
	Relevance	11	4.3	1.0	5	4–5	0	1	1	3	6
	Usability	11	4.0	1.0	4	3.5–5	0	1	2	4	4
Metric Dominance	Clarity	11	4.4	0.7	4	4–5	0	0	1	5	5
	Relevance	11	3.9	1.0	4	4–4.5	0	2	0	6	3
	Usability	11	3.9	0.8	4	4–4	0	1	1	7	2
Disciplinary Divides	Clarity	11	4.4	0.8	5	4–5	0	0	2	3	6
	Relevance	11	4.2	0.9	4	4–5	0	1	0	6	4
	Usability	11	3.9	0.9	4	3.5–4.5	0	1	2	5	3

No rating of 1 occurred among the 722 valid CRU ratings. All item-level medians were 4 or 5.
